# Comparative Analysis of Six *Lagerstroemia* Complete Chloroplast Genomes

**DOI:** 10.3389/fpls.2017.00015

**Published:** 2017-01-19

**Authors:** Chao Xu, Wenpan Dong, Wenqing Li, Yizeng Lu, Xiaoman Xie, Xiaobai Jin, Jipu Shi, Kaihong He, Zhili Suo

**Affiliations:** ^1^State Key Laboratory of Systematic and Evolutionary Botany, Institute of Botany, Chinese Academy of SciencesBeijing, China; ^2^University of Chinese Academy of SciencesBeijing, China; ^3^Peking-Tsinghua Center for Life Sciences, Academy for Advanced Interdisciplinary Studies, Peking UniversityBeijing, China; ^4^Shandong Provincial Center of Forest Tree Germplasm ResourcesJinan, China; ^5^Beijing Botanical Garden, Institute of Botany, Chinese Academy of SciencesBeijing, China; ^6^Xishuangbanna Tropical Botanical Garden, Chinese Academy of SciencesMengla, China

**Keywords:** *Lagerstroemia*, chloroplast genome, comparative genomics, simple repeat sequence, sequence divergence, plastid marker, phylogeny

## Abstract

Crape myrtles are economically important ornamental trees of the genus *Lagerstroemia L*. (Lythraceae), with a distribution from tropical to northern temperate zones. They are positioned phylogenetically to a large subclade of rosids (in the eudicots) which contain more than 25% of all the angiosperms. They commonly bloom from summer till fall and are of significant value in city landscape and environmental protection. Morphological traits are shared inter-specifically among plants of *Lagerstroemia* to certain extent and are also influenced by environmental conditions and different developmental stages. Thus, classification of plants in *Lagerstroemia* at species and cultivar levels is still a challenging task. Chloroplast (cp) genome sequences have been proven to be an informative and valuable source of cp DNA markers for genetic diversity evaluation. In this study, the complete cp genomes of three *Lagerstroemia* species were newly sequenced, and three other published cp genome sequences of *Lagerstroemia* were retrieved for comparative analyses in order to obtain an upgraded understanding of the application value of genetic information from the cp genomes. The six cp genomes ranged from 152,049 bp (*L. subcostata*) to 152,526 bp (*L. speciosa*) in length. We analyzed nucleotide substitutions, insertions/deletions, and simple sequence repeats in the cp genomes, and discovered 12 relatively highly variable regions that will potentially provide plastid markers for further taxonomic, phylogenetic, and population genetics studies in *Lagerstroemia*. The phylogenetic relationships of the *Lagerstroemia* taxa inferred from the datasets from the cp genomes obtained high support, indicating that cp genome data may be useful in resolving relationships in this genus.

## Introduction

On the earth, some major subclades (i.e., Rosids, Asterids, Saxifragales, Santalales, and Caryophyllales) are recognized phylogenetically under the eudicot clade of angiosperms, consisting of ~75% of all flowering plant species. Among the subclades, the rosids are grouped together as a large evolutionary monophyletic group, containing more than 25% of all angiosperms. *Lagerstroemia* plants are positioned phylogenetically in the Lythraceae (within the Myrtales Rchb.) of the rosids among core eudicots. *Lagerstroemia*, one of the 25 genera in the family Lythraceae, has about 56 species in the world, with a distribution from the tropical to northern temperate zones (Qin and Shirley, 2007; APG III, 2009; Su et al., 2014).

Crape myrtles produce abundant large and beautiful panicles with charming flowers commonly lasting for about 3 months or more across summer and autumn seasons (Qin and Shirley, 2007). Their leaves can clean the air by absorbing smoke and dust. They are well-known excellent ornamental trees for city gardening and environmental protection. Their cultivation has a history of at least 1500 years in China. At present, more than 500 cultivars have been bred in the world. They have significant value in horticultural and landscaping application (Huang et al., 2013a,b,c).

Phylogenetic relationships within Lythraceae have been approached using morphology and DNA evidences from the *rb*cL gene, the *trn*L-F region, and the *psa*A-*ycf* 3 intergenic spacer of the cp genome, and ITS (the internal transcribed spacer) of the nuclear genome (Huang and Shi, 2002; Graham et al., 2005). The four DNA markers (*rbc*L, *mat*K, *trn*H-*psb*A, and ITS) can only meet the need for plant identification at/above species level with limited or no resolution among closely related species and/or cultivars (Xiang et al., 2011; Suo et al., 2012, 2015, 2016). Due to shared morphological traits to some extent among species and cultivars, the lack of morphological and DNA markers heavily inhibited the genetic diversity evaluation of *Lagerstroemia* germplasm resources. Genetic information from comparative genomics for researches on genetic diversity and phylogeny in the *Lagerstroemia* is limited (Pounders et al., 2007; Wang et al., 2011; Suo et al., 2012, 2015, 2016; He et al., 2014; Gu et al., 2016a,b).

Chloroplasts are key organelles in plants for photosynthesis and other biochemical pathways such as the biosynthesis of starch, fatty acids, pigments, and amino acids (Dong et al., 2013, 2016; Raman and Park, 2016). Chloroplast (cp) genome, as one of the three DNA genomes (the other two are nuclear and mitochondrial genomes) in plant body, with uniparental inheritance, has a highly conserved circular DNA arrangement ranging from 115 to 165 kb. Complete cp genome sequences have been widely accepted as an informative and valuable data source for understanding evolutionary biology because of their relatively stable genome structure, gene content, and gene order (Dong et al., 2012, 2013, 2014, 2016; Suo et al., 2012, 2015, 2016; Curci et al., 2015; Downie and Jansen, 2015; Song et al., 2015). Along with the accumulation of complete cp genome sequences, comparative study of chloroplast genomes from *Lagerstroemia* plants is helpful for upgrading our evaluation on the application value of the cp genomes.

In this study, we report three newly sequenced complete cp genomes from the *Lagerstroemia* (two species and one cultivar) and genomic comparative analyses with other three published cp genome sequences of the genus downloaded from the National Center for Biotechnology Information (NCBI) organelle genome database (https://www.ncbi.nlm.nih.gov), focusing on organization, gene content, patterns of nucleotide substitutions, and simple sequence repeats (SSRs). The aims of our study are: (i) to deepen our understanding on the genetic and evolutionary significance from the structural diversity in the cp genomes, (ii) to upgrade our understanding on the application value of the complete cp genomes of *Lagerstroemia*, and (iii) to provide genetic resources for future research in this genus.

## Materials and methods

### Plant materials and DNA extraction

Fresh leaves were collected from the trees of *Lagerstroemia subcostata* and *L. indica* “Lüzhao Hongdie” growing in the Beijing Botanical Garden (N 39°48′, E 116°28′, Altitude 76 m) of the Chinese Academy of Sciences, and from the trees of *L. speciosa* growing in the Xishuangbanna Tropical Botanical Garden (N 21°41′, E 101°25′, Altitude 570 m), the Chinese Academy of Sciences. The fresh leaves from each accession were immediately dried with silica gel for further DNA extraction. Total genomic DNAs were extracted from each sample using the Plant Genomic DNA Kit (DP305) from Tiangen Biotech (Beijing) Co., Ltd., China.

### Chloroplast genome sequencing, assembling, and annotation

The *Lagerstroemia* cp genomes were sequenced using the short-range PCR (Polymerase Chain Reaction) method reported by Dong et al. (2012, 2013). The PCR protocol was as follows: preheating at 94°C for 4.5 min, 34 cycles at 94°C for 50 s, annealing at 55°C for 40 s, and elongation at 72°C for 1.5 min, followed by a final extension at 72°C for 8 min. PCR amplification was performed in an Applied Biosystems VeritiTM 96-Well Thermal Cycler (Model#: 9902, made in Singapore). The amplified DNA fragments were sent to Shanghai Majorbio Bio-Pharm Technology Co., Ltd (Beijing) for Sanger sequencing in both the forward and reverse directions using a 3730xl DNA analyzer (Applied Biosystems, Foster City, CA, USA). DNA regions containing poly structures or difficult to amplify were further sequenced using newly designed primers for confirming reliable and high quality sequencing results.

**Figure 1:**
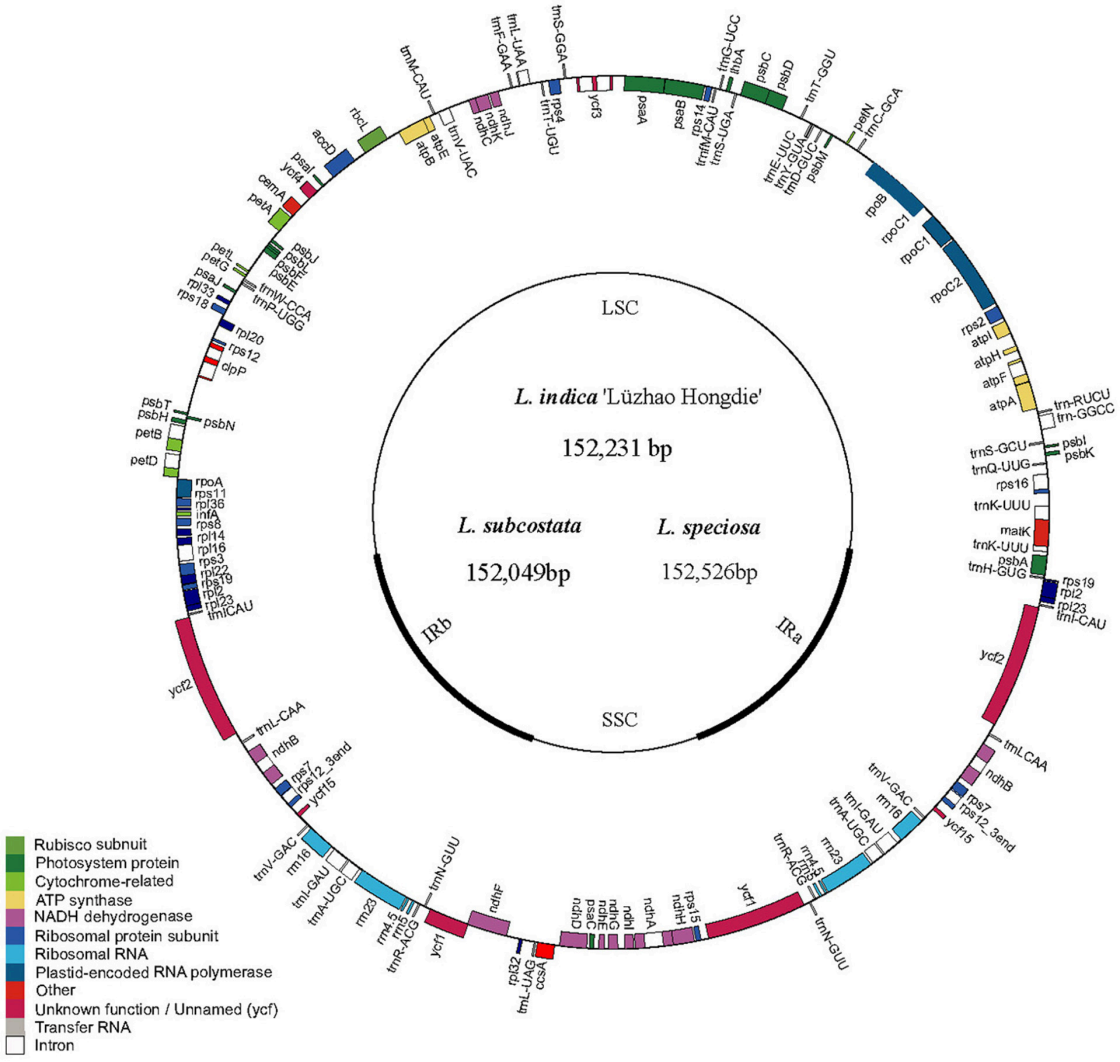
**Gene map of ***Lagerstroemia*** chloroplast genome**. The genes inside and outside of the circle are transcribed in the clockwise and counterclockwise directions, respectively. Genes belonging to different functional groups are shown in different colors. The thick lines indicate the extent of the inverted repeats (IRa and IRb) that separate the genomes into small single-copy (SSC) and large single-copy (LSC) regions.

The cp DNA sequences were manually confirmed and assembled using Sequencher (v4.6) software, and cp genome annotation was performed using the Dual Organellar Genome Annotator (DOGMA; Wyman et al., 2004). BLASTX and BLASTN searches were employed to accurately annotate the protein-encoding genes and to identify the locations of the ribosomal RNA (rRNA) and transfer RNA (tRNA) genes. Gene annotation information from other closely related plant species was also utilized for confirmation when the boundaries of the exons or introns could not be precisely determined because of the limited power of BLAST in cp genome annotation. The cp genome map was drawn using Genome Vx software (Conant and Wolfe, 2008; Figure 1). The cp genome sequences have been deposited to GenBank with the following accession numbers: KF572028 for *L. indica* “Lüzhao Hongdie,” KF572029 for *L. subcostata* and KX572149 for *L. speciosa*. The cp genome sequences of *L. fauriei* (KT358807), *L. indica* (KX263727), and *L. guilinensis* (KU885923) were downloaded from GenBank (https://www.ncbi.nlm.nih.gov).

### Simple sequence repeat analysis

Perl script MISA (Thiel et al., 2003) was used to search for simple sequence repeat (SSRs or microsatellites) loci in the cp genomes. The minimum numbers (thresholds) of the SSRs were 10, 5, 4, 3, 3, and 3 for mono-, di-, tri-, tetra-, penta-, and hexa-nucleotides, respectively. All of the repeats found were manually verified and redundant results were removed.

### Chloroplast genome analysis by sliding window

**Figure 2:**
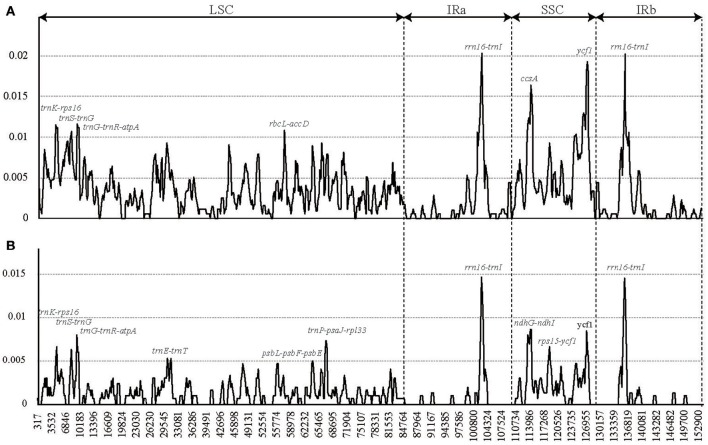
**Sliding window analysis of the whole chloroplast genomes of six ***Lagerstroemia*** taxa (A)** and five *Lagerstroemia* taxa (not including *L. speciosa*) **(B)** (window length: 600 bp, step size: 200 bp). X-axis, position of the midpoint of a window; Y-axis, nucleotide diversity of each window.

These cp genome sequences were aligned using MAFFT (Katoh and Standley, 2013) and were manually adjusted using Se-Al 2.0 (Rambaut, 1996). We used two data sets (the sequence alignment of all the six complete *Lagerstroemia* cp genomes and the sequence alignment of five *Lagerstroemia* cp genomes excluding *L. speciosa*) for sliding window analysis, because of the high divergence of *L. speciosa* from the other five cp genomes (Figure 2). Sliding window analysis was conducted to generate nucleotide diversity (Pi) of the cp genome using DnaSP (DNA Sequences Polymorphism version 5.10.01) software (Librado and Rozas, 2009). The step size was set to 200 bp, with a 600 bp window length.

### Sequence divergence analysis

**Figure 3:**
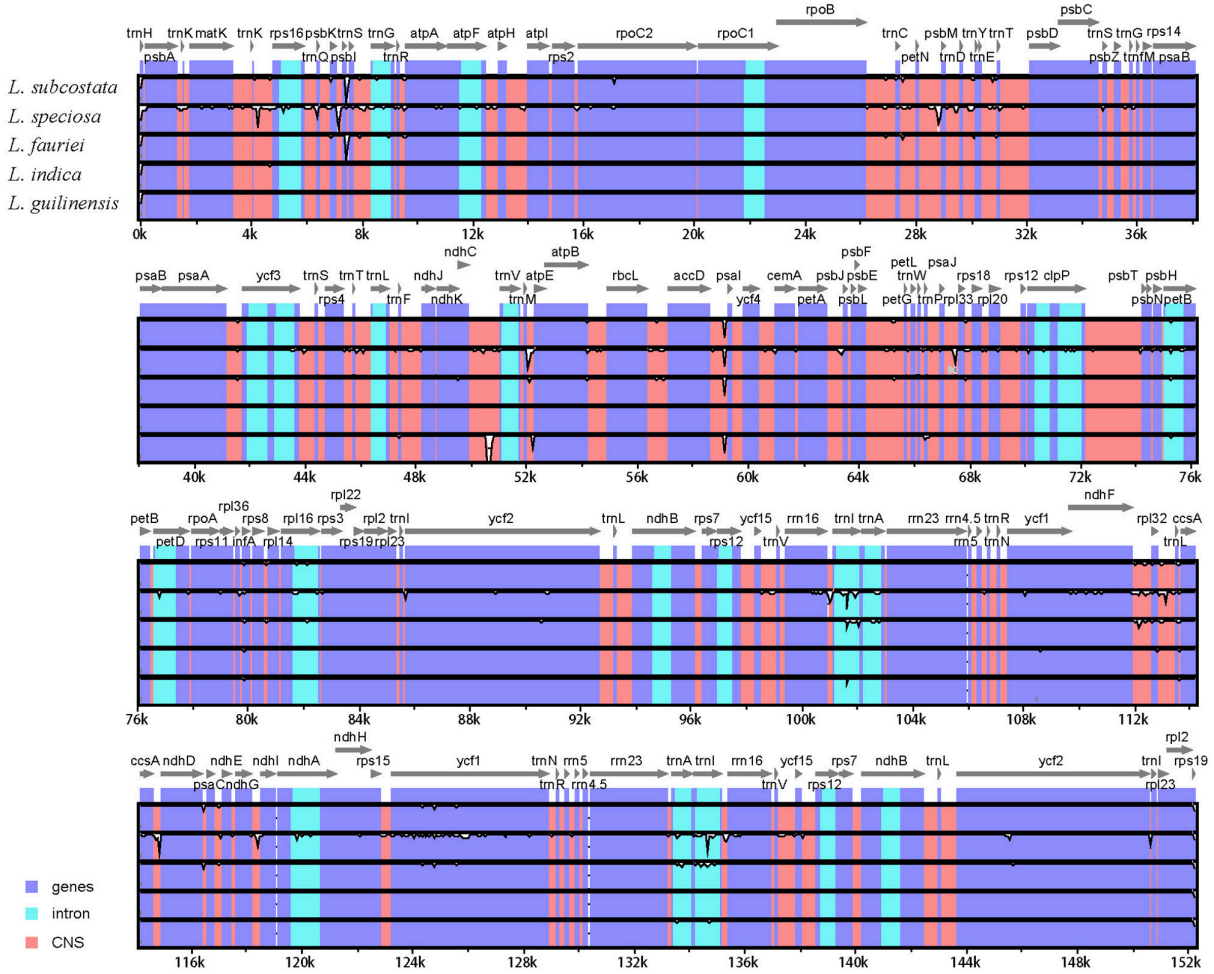
**Identity plot comparing the chloroplast genomes of six ***Lagerstroemia*** taxa using ***L. indica*** “Lüzhao Hongdie” as a reference sequence**. The vertical scale indicates the percentage of identity, ranging from 50 to 100%. The horizontal axis indicates the coordinates within the chloroplast genome. Genome regions are color coded as protein-coding, rRNA, tRNA, intron, and conserved non-coding sequences (CNS).

The alignment of the six *Lagerstroemia* complete cp genome sequences was visualized using mVISTA program in Shuffle-LAGAN mode (Frazer et al., 2004) in order to show inter- and intra-specific variations (Figure 3). Variable and parsimony-informative base sites across the complete cp genomes, and the large single copy (LSC), small single copy (SSC), and inverted repeats (IR) regions of the six cp genomes were calculated using Mega 6.0 software (Tamura et al., 2013). Insertions/deletions (indels) were manually detected using DnaSP software. To estimate selection pressures, non-synonymous (dN), and synonymous (dS) substitution rates of the combined sequences of 79 protein coding genes were calculated using PAML with the yn00 program (Yang, 2007).

### Phylogenetic analysis

**Figure 4:**
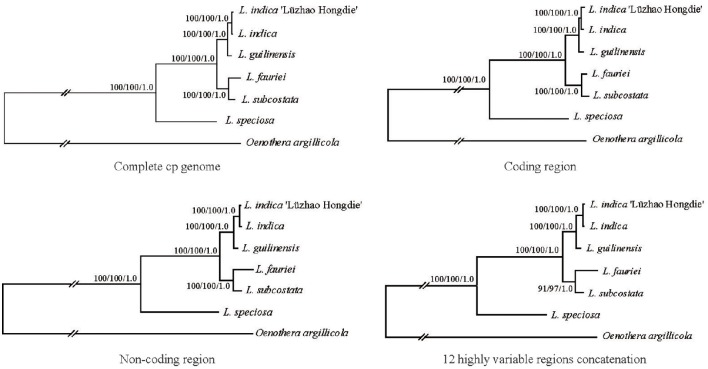
**Phylogenetic relationships of the six ***Lagerstroemia*** taxa constructed by each of the four DNA sequence alignment data sets including whole cp genome sequences, coding regions, non-coding regions, and the 12 highly variable regions concatenation with maximum parsimony (MP), maximum likelihood (ML), and Bayesian inference (BI) methods**. ML topology shown with MP bootstrap support values/ML bootstrap support value/Bayesian posterior probability listed at each node.

Phylogenetic analysis was conducted using the complete chloroplast genome sequences of the six *Lagerstroemia* taxa mentioned above, with one Onagraceae species (*Oenothera argillicola*, 165,061 bp, GenBank accession No. EU262887) that was used as an outgroup (Figure 4).

Maximum parsimony (MP) analyses were conducted using PAUP v4b10 (Swofford, 2003). All characters were equally weighted, gaps were treated as missing, and character states were treated as unordered. Heuristic search was performed with MULPARS option, tree bisection-reconnection (TBR) branch swapping, and random stepwise addition with 1,000 replications. The Maximum likelihood (ML) analyses were performed using RAxML 8.0 (Stamatakis, 2006). For ML analyses, the best-fit model, general time reversible (GTR)+G was used in all analysis as suggested with 1,000 bootstrap replicates.

Bayesian inference (BI) was performed with Mrbayes v3.2 (Ronquist et al., 2012). The Markov chain Monte Carlo (MCMC) analysis was run for 2 × 5,000,000 generations. Trees were sampled at every 1,000 generations with the first 25% discarded as burn-in. The remaining trees were used to build a 50% majority-rule consensus tree. The stationarity was considered to be reached when the average standard deviation of split frequencies remained below 0.001.

## Results and discussion

### Chloroplast genome organization of the *Lagerstroemia* taxa

The nucleotide sequences of the six *Lagerstroemia* cp genomes ranged from 152,049 bp (*L. subcostata*) to 152,526 bp (*L. speciosa*) in length (Figure 1 and Table 1). The six *Lagerstroemia* cp genome sequences have minor differences in length (no more than 477 bp; Table 1). The average GC content was 37.59%, which is almost identical with each other among the six complete *Lagerstroemia* cp genomes. When duplicated genes in IR regions were counted only once, the six *Lagerstroemia* cp genomes each identically harbored 112 different genes with the same arrangement order, including 78 protein-coding, 4 rRNA, and 30 tRNA genes (Figure 1, Table 1, and Table S1). The gene organization, gene order and GC content were highly identical and similar to those of other higher plants (Figure 1). The overall genomic structure including gene number and gene order were well-conserved.

**Table 1:** **Summary of complete chloroplast genome features of the six ***Lagerstroemia*** taxa**.

	***L. indica* “Lüzhao Hongdie”**	***L. indica***	***L. subcostata***	***L. speciosa***	***L. fauriei***	***L. guilinensis***
Large single copy (LSC, bp)	84,062	84,046	83,890	84,193	83,920	83,811
Small single copy (SSC, bp)	16,919	16,915	16,909	16,833	16,934	16,909
Inverted repeat (IR, bp)	25,625	25,622	25,625	25,750	25,793	25,677
Total	152,231	152,205	152,049	152,526	152,440	152,074
Protein-coding genes	78	78	78	78	78	78
rRNA	4	4	4	4	4	4
tRNA	30	30	30	30	30	30
Total	112	112	112	112	112	112
GC%	37.59	37.59	37.59	37.57	37.60	37.62

Although cp genomes are highly conserved in terms of genomic structure and size, the IR/SC junction position change caused by expansion and contraction of the IR/SC boundary regions was usually considered as a primary mechanism in creating the length variation of the higher plant cp genomes (Kim and Lee, 2005; Asaf et al., 2016; Dong et al., 2016; Yang et al., 2016; Zhang et al., 2016). In this study, however, the IR/SC junction position change was not observed among the six cp genomes. This indicated that the IR/SC junction is relatively conserved in *Lagerstroemia* in comparison with other plant groups, such as *Quercus* (Yang et al., 2016) and *Epimedium* (Zhang et al., 2016). Further, study is necessary by sampling more species of the genus across the world for confirmation.

The *rpl2* intron loss was observed in the three newly sequenced *Lagerstroemia* cp genomes in this study. The occurrence of *rpl2* intron loss in *Lagerstroemia* was considered to be one of the important evolutionary events in the Lythraceae of the rosids. It was inferred to occur after the divergence of the Lythraceae from the Onagraceae, but prior to the divergence of the Lythraceae genera (Gu et al., 2016a).

### SSR analysis of the *Lagerstroemia* cp genomes

Simple sequence repeats (SSRs) in the cp genome can be highly variable at the intra-specific level, and are therefore often used as genetic markers in population genetics and evolutionary studies (Dong et al., 2013, 2016; Kaur et al., 2015; Suo et al., 2016; Yang et al., 2016). We analyzed the simple sequence repeats (SSRs) in the cp genomes (Tables 2, 3, Tables S2, S3). The lengths of SSRs ranged from 10 to 15 bp. Comparative analysis of the six *Lagerstroemia* cp genome sequences indicated that totally five categories of SSRs (mono-nucleotide, di-nucleotide, tri-nucleotide, tetra-nucleotide, and penta-nucleotide repeats) were detected, including 35 SSR types and 275 SSR loci. The most abundant were mono-nucleotide repeats, which accounted for 53.82% in the total, followed by tetra-nucleotide repeats (16.36%), tri-nucleotide repeats (14.91%), and di-nucleotides repeats (10.55%), subsequently. Penta-nucleotide repeats had the least amount (4.36%; Tables 2, 3, Tables S2, S3). In *Quercus* species, mononucleotide repeats are the most abundant, accounting for about 80% of the total SSRs (Yang et al., 2016). In the cp genome of *Dianthus*, homopolymers were most common, accounting for 95.58% of the SSRs (Raman and Park, 2015). These results suggest that mononucleotide repeats may contribute more to the genetic variations in comparison with other SSRs. The SSR information will be important for understanding the genetic diversity status of the global *Lagerstroemia* plants.

**Table 2:** **Distribution of each SSR category in the six ***Lagerstroemia*** cp genomes**.

	**Category**	**Number**	**Intergenic**	**Gene**	**Intron**	**LSC**	**SSC**	**IRa**	**IRb**
*L. fauriei*	Mono-nucleotide	28	18	4	6	28	6	2	2
	Di-nucleotide	4	1	2	1	3	1	0	0
	Tri-nucleotide	6	4	2	1	3	2	1	1
	Tetra-nucleotide	7	3	3	2	6	2	0	0
	Penta-nucleotide	2	2	0	0	0	0	1	1
	Subtotal	47	28	11	10	40	11	4	4
*L. guilinensis*	Mono-nucleotide	24	17	4	3	16	5	2	2
	Di-nucleotide	4	2	2	1	4	1	0	0
	Tri-nucleotide	7	4	2	1	3	2	1	1
	Tetra-nucleotide	7	2	3	2	5	2	0	0
	Penta-nucleotide	2	2	0	0	0	0	1	1
	Subtotal	44	27	11	7	28	10	4	4
*L. indica*	Mono-nucleotide	18	10	3	5	11	5	1	1
	Di-nucleotide	6	4	1	1	4	1	1	0
	Tri-nucleotide	7	4	2	1	3	2	1	1
	Tetra-nucleotide	7	2	3	2	5	2	0	0
	Penta-nucleotide	2	2	0	0	0	0	1	1
	Subtotal	40	22	9	9	23	10	4	3
*L. indica* “Lüzhao Hongdie”	Mono-nucleotide	29	19	4	6	18	7	2	2
	Di-nucleotide	5	3	2	0	4	1	0	0
	Tri-nucleotide	7	4	2	1	3	2	1	1
	Tetra-nucleotide	7	2	3	2	5	2	0	0
	Penta-nucleotide	2	2	0	0	0	0	1	1
	Subtotal	50	30	11	9	30	12	4	4
*L. speciosa*	Mono-nucleotide	24	17	2	5	20	2	1	1
	Di-nucleotide	6	3	2	1	4	2	0	0
	Tri-nucleotide	7	4	2	1	4	1	1	1
	Tetra-nucleotide	9	4	3	2	7	2	0	0
	Penta-nucleotide	2	2	0	0	0	0	1	1
	Subtotal	48	30	9	9	35	7	3	3
*L. subcostata*	Mono-nucleotide	25	15	4	6	15	6	2	2
	Di-nucleotide	4	1	2	1	3	1	0	0
	Tri-nucleotide	7	4	2	1	3	2	1	1
	Tetra-nucleotide	8	3	4	1	6	2	0	0
	Penta-nucleotide	2	1	0	1	0	0	1	1
	Subtotal	46	24	12	10	27	11	4	4
	Total	275	161	63	54	183	61	23	22

**Table 3:** **Numbers and percentage of SSRs in the six ***Lagerstroemia*** cp genomes**.

**Taxa**	**Number of SSRs (Percentage in the total) in different regions of the cp genomes**							**Total**
	**Intergenic**	**Gene**	**Intron**	**LSC**	**SSC**	**IRa**	**IRb**	
*L. fauriei*	28 (59.57%)	11 (23.40%)	10 (21.28%)	40 (85.11%)	11 (23.40%)	4 (8.51%)	4 (8.51%)	47
*L. guilinensis*	27 (61.36%)	11 (25.00%)	7 (15.91%)	28 (63.64%)	10 (22.73%)	4 (9.09%)	4 (9.09%)	44
*L. indica*	22 (55.00%)	9 (22.50%)	9 (22.50%)	23 (57.50%)	10 (25.00%)	4 (10.00%)	3 (7.50%)	40
*L. indica* “Lüzhao Hongdie”	30 (60.00%)	11 (22.00%)	9 (18.00%)	30 (60.00%)	12 (24.00%)	4 (8.00%)	4 (8.00%)	50
*L. speciosa*	30 (62.50%)	9 (18.75%)	9 (18.75%)	35 (72.92%)	7 (14.58%)	3 (6.25%)	3 (6.25%)	48
*L. subcostata*	24 (52.17%)	12 (26.09%)	10 (21.74%)	27 (58.70%)	11 (23.91%)	4 (8.70%)	4 (8.70%)	46
Average	26.8	10.5	9.0	30.5	10.2	3.8	3.7	45.8
Min.–Max.	22–30	9–12	7–10	23–40	7–12	3–4	3–4	40–50
Total	161 (58.55%)	63 (22.91%)	54 (19.64%)	183 (66.55%)	61 (22.18%)	23 (8.36%)	22 (8.00%)	275

*SSRs, simple sequence repeats. LSC, Large single copy region; SSC, Small single copy region; Ira, Inverted repeat region a; IRb, Inverted repeat region b*.

In this study, these 275 SSRs were mainly located in intergenic spacers (161 SSRs, 58.55%) or LSC region (183 SSRs, 66.55%), only a minority (IRa: 22 SSRs, 8.00%; IRb: 23 SSRs, 8.36%) of SSRs were located in the IR regions. Sixty-three SSRs (22.91%) were located in eight gene (CDS) regions (*rpoA, rpoB, rpoC2, cemA, ndhD, ndhF, ycf1, ycf2*; Tables 2, 3, Tables S2, S3). Fifty-four SSRs (19.64%) were located in intron regions. The distribution of SSRs is variable significantly among the four regions in each of the six *Lagerstroemia* cp genomes, which is identical with previous reports (Dong et al., 2016; Yang et al., 2016).

Among the 148 homopolymer SSRs of the six *Lagerstroemia* cp genomes, 141 (95.27%) are the A/T type, distributed mostly in intergenic (90 A/T loci, 63.83%) and LSC (102 A/T loci, 72.34%) regions (Tables S2, S3). In *Nicotiana otophora*, all mono-nucleotides (100%) are composed of A/T (Asaf et al., 2016). In the five *Epimedium* cp genomes, mono-nucleotide SSRs were found to be the richest, up to 72.76%, and the mono-nucleotide A/T repeat units occupied 80.17% in the homopolymer SSRs. Our results are identical with the observation that the occurrence of transversion substitutions is correlated to some extent with high A/T content regions of the cp genome (Morton and Clegg, 1995; Morton et al., 1997).

In the cp genomes of five *Quercus* species, most of the repeat units were distributed in intergenic or intron regions, and only a minority were located in gene regions (*ycf1, ycf2, psaA, psaB, trnS-GCU, trnS-UGA, trnG-GCC, trnG-UCC, trnS-UGA*, and *trnS-GGA*; Yang et al., 2016).

In this study, no variation was detected in the repeat number of penta-nucleotide repeat category and only minor variation was observed in the repeat number of tri-nucleotide repeat category among species and/or cultivars. The repeat numbers of mono-nucleotide, di-nucleotide and tetra-nucleotide repeat categories were found variable significantly among the six cp genomes. Mono-nucleotide repeat category is the dominant variation source, especially between cultivars rather than between species, e.g., with 29 in *L. indica* “Lüzhao Hongdie,” and 18 in *L. indica* (Tables 2, 3, Tables S2, S3).

In the five *Epimedium* cp genomes, the detected 116 SSR loci mainly located in intergenic spacers (IGS, 62.07%), followed by introns (23.28%) and CDS (13.79%) regions. These are similar with our results. It was observed that 16 SSRs were located in 10 protein-coding genes (*rpoC2, rpoB, psbC, psaA, psbF, ycf1, ycf2, rpl32, ndhE*, and *ndhH*) of the five *Epimeidium* cp genomes (Zhang et al., 2016). Therefore, evidences strongly suggest that the occurrence and genetic variations of SSRs in genes (such as, *ycf* 1) may have phylogenetic significance. This is worth further study in the future.

A preference for occurrence of SSRs in intergenic or gene regions was observed between plant families and among the samples/taxa within family. The cp SSRs of the six *Lagerstroemia* taxa represented abundant variation, and are useful for detecting genetic polymorphisms at population, intraspecific, and cultivar levels as well as comparing more distant phylogenetic relationships among *Lagerstroemia* species.

### Genome sequence divergence among the *Lagerstroemia* species/cultivars

We used mVISTA to perform a sequence identity analysis, with *L. indica* “Lüzhao Hongdie” as a reference (Figure 3). The alignment revealed high sequence similarity across the cp genomes, which suggests that they are highly conserved. Non-coding and SC regions exhibit higher divergence levels than coding and IR regions, respectively.

The LSC and SSC regions contributed 150 and 55 informative base sites, respectively, while the IR regions contributed only 15 informative sites (Table 4). The SSC region showed the highest nucleotide diversity (0.00639), followed by the LSC region (0.00345) and the IR region (0.00175; Table 4). *Lagerstroemia speciosa* presented the highest numbers of nucleotide substitutions and insertions/deletions (indels) among the six *Lagerstroemia* taxa, while the nucleotide diversity, and the numbers of nucleotide substitutions and insertions/deletions (indels) at cultivar level were found to be the smallest (Tables 4, 5).

**Table 4:** **Variable site analyses in the six ***Lagerstroemia*** cp genomes**.

	**Number of sites**	**Number of variable sites**	**Number of informative sites**	**Nucleotide diversity**
Large single copy region	84,868	771 (0.91%^*^)	150 (19.46%^**^)	0.00345
Small single copy region	17,077	281 (1.65%)	55 (19.57%)	0.00639
Inverted repeat region	25,961	133 (0.51%)	15 (11.28%)	0.00175
Complete cp genome	153,842	1330 (0.86%)	238 (17.89%)	0.00322

* *The percentage of variable sites in the number of sites*.

** *The percentage of informative sites in the number of variable sites*.

**Table 5:** **Number of nucleotide substitutions and insertions/deletions in the six ***Lagerstroemia*** complete cp gemomes**.

	***L. indica* “Lüzhao Hongdie”**	***L. subcostata***	***L. speciosa***	***L. fauriei***	***L. indica***	***L. guilinensis***
*L. indica* “Lüzhao Hongdie”		66	293	95	29	31
*L. subcostata*	257		295	57	79	72
*L. speciosa*	1084	1089		315	297	301
*L. fauriei*	309	134	1105		103	91
*L. indica*	24	249	1082	303		44
*L. guilinensis*	63	254	1083	291	57	

*The lower triangle indicates the number of nucleotide substitutions, the upper triangle shows the number of insertions/deletions*.

Pairwise substitution rates (dN/dS) between the *Lagerstroemia* cp genomes were calculated based on the 78 protein-coding gene sequences (Table 6). The numbers of nucleotide substitutions and indels varied from 29 to 315, and 24 to 1089, respectively (Table 5). There were always fewer dN than dS. The dN/dS ratio ranged from 0.1688 to 0.6081. The highest dN/dS ratio occurred between *L. indica* and *L. guilinensis*. The lowest dN/dS ratio occurred between *Lagerstroemia indica* and *L. indica* “Lüzhao Hongdie” (Table 6). In our study, the dN/dS ratio is below 1, indicating that the related gene regions might be under negative selection.

**Table 6:** **Pairwise substitution rates (dN/dS) between the ***Lagerstroemia*** chloroplast genomes based on the 78 protein-coding gene sequences**.

**No**.	***L. indica* “Lüzhao Hongdie”**	***L. subcostata***	***L. speciosa***	***L. fauriei***	***L. indica***
*L. indica* “Lüzhao Hongdie”					
*L. subcostata*	0.3102 (0.0009/0.0029)				
*L. speciosa*	0.3762 (0.0036/0.0095)	0.3755 (0.0037/0.0098)			
*L. fauriei*	0.3178 (0.0009/0.0027)	0.2605 (0.0002/0.0009)	0.3710 (0.0036/0.0097)		
*L. indica*	0.1688 (0.0001/0.0006)	0.3374 (0.0010/0.0028)	0.3767 (0.0036/0.0094)	0.3326 (0.0009/0.0027)	
*L. guilinensis*	0.3420 (0.0002/0.0005)	0.3174 (0.0009/0.0028)	0.3711 (0.0035/0.0094)	0.2963 (0.0008/0.0026)	0.6081 (0.0002/0.0003)

We chose the 12 relatively highly variable regions including 2 gene regions and 10 intergenic regions from the cp genomes that might be undergoing a more rapid nucleotide substitution at species and cultivar levels, as potential molecular markers for application in phylogenetic analyses and plant identification in *Lagerstroemia* (Figure 2, Table 7). They are *trnK-rps16, trnS-trnG, trnG-trnR-atpA, trnE-trnT, rbcL-accD, psbL-psbF-psbE, trnP-psaJ-rpl33, rrn16-trnI, ccsA, ndhG-ndhI, rps15-ycf1*, and *ycf1*. Primers for these regions are shown in Table 7. Yang et al. (2016) determined five most variable coding regions and 14 most variable non-coding regions as potential molecular markers for *Quercus* germplasm resources, which are identical with the variable regions found in *Lagerstroemia*, except for *trnE-trnT, psbL-psbF-psbE, trnP-psaJ-rpl33, ndhG-ndhI*, and *rps15-ycf1*. Further, study is expected to utilize these cp DNA markers in global detection of the *Lagerstroemia* germplasm resources.

**Table 7:** **Primers for PCR amplification of the 12 relatively highly variable regions among the six ***Lagerstroemia*** taxa**.

**No**.	**Region amplified**	**Forward primer (5′ → 3′)**	**Reverse primer (5′ → 3′)**	**Size (bp)**	**Annealing temperature (°C)**
1	*trn*K-*rps*16	TGGGTTCATAGGACTCTATCCA	TTGCAATTGATGTGCGATCTCGA	1202	56
2	*trn*S-*trn*G	ACCGAGTTATCAACGGAAACGGA	TAAAGTTTCTGCTCGGAATAAGA	882	53
3	*trn*G-*trn*R-*atp*A	TCTAGAGGGATTATCTAGAAAGCA	AAGAGGTCAACGATTACGTGAGT	975	55
4	*trn*E-*trn*T	AGAGGAATGTCCGTTGGG	CGATGACTTACGCCTTACC	1471	53
5	*rbc*L-*acc*D	TCTCTTAATTGAATTGCAATTCA	AATAGATGAATAGTCATTCGATGA	703	49.5
6	*psb*L-*psb*F-*psb*E	GTGATCCTTCCGAATGGGATAAG	CAGTGAATTTCCATTTACTGATAT	672	51.8
7	*trn*P-*psa*J-*rpl*33	AGTAGAAGGTTTATATATCTAATA	GATTATTTCGTTGCAATCACAAC	905	48.5
8	*rrn*16-*trn*I	TTAGTTGCCACCGGTATGAGAGT	GGTCCTCTTCCCCATTACTTAGA	1845	58
9	*ccs*A	AGGTATAATCCATGAATATTGAT	TGAATTCATTATAGGACTTATTA	1755	48
10	*ndh*G-*ndh*I	ATCGGTTGATAAATGAATTCCAA	CAAGGTTCAATTTGATCTAATCT	790	51
11	*rps*15-*ycf*1	TAAGTCTTCGTATCTTATTGGTG	GAGTTTGGATATTCTGATGATTCA	1122	53
12	*ycf*1	TAACCTCAGCCTTAGCATT	GGACAGAATAGACAAACCCT	2191	50

### Phylogenetic analysis

Phylogenetic analysis using cp genome sequences have resolved numerous lineages within the flowering plants (Jansen et al., 2007; Moore et al., 2007). The cp DNA regions of *atpF-atpH, matK, psbK-psbI, rbcL*, and *trnH-psbA* have been recommended and used as species-level barcodes with a great success (Suo et al., 2012, 2015, 2016; Dong et al., 2015, 2016). However, these five cp DNA markers are not powerful enough when closely related species or cultivars are under considerations. Therefore, genomic comparative researches of more complete cp genome sequences have become necessary.

In this study, all of the six *Lagerstroemia* taxa were discriminated completely with high bootstrap support based on each of the four DNA sequence alignment data sets including whole cp genome sequences, coding regions, non-coding regions, and the 12 highly variable regions concatenation using maximum parsimony (MP), maximum likelihood (ML), and Bayesian inference (BI) methods (Figure 4). *L. guilinensis, L. indica* “Lüzhao Hongdie,” and *L. indica* showed a very close genetic relationship. The six taxa were separated into three evolutionary branches. The branch including *L. subcostata* and *L. fauriei* was a sister to the branch containing *L. guilinensis, L. indica* “Lüzhao Hongdie,” and *L. indica*. *L. speciosa* was placed at the basal position, and showed a large divergence from the rest five *Lagerstroemia* taxa. A better resolution was obtained by the sequence data set from the non-coding regions as compared to each of the other three datasets. Similar resolution can be obtained using a sequence data set from 12 highly variable cp regions with lower cost.

## Conclusions

This study reports the comparative analysis results of six *Lagerstroemia* cp genome sequences with detailed gene annotation. The six cp genomes are similar in structure and have a high degree of the synteny of gene order. The IR/SC junction position change was not observed among the six cp genomes, indicating that the IR/SC junction is relatively conservative in *Lagerstroemia* in comparison with other plant groups, such as *Quercus* and *Epimedium*. Further study is necessary for confirmation within the whole genus by sampling more species. Twelve cp DNA markers were developed from the relatively highly variable regions. All of the six *Lagerstroemia* taxa were discriminated completely with high bootstrap support based on each of the four DNA sequence alignment data sets including whole cp genome sequences, coding regions, non-coding regions, and 12 highly variable regions using maximum parsimony (MP), maximum likelihood (ML), and Bayesian inference (BI) methods. A better resolution was obtained by the sequence data set from the non-coding regions rather than by each of the other three data sets, with no significant difference among the analytic methods. Similar resolution result can be obtained by the sequence data set from 12 highly variable regions with lower cost. The six taxa were separated into three evolutionary branches. The branch including *L. subcostata* and *L. fauriei* is a sister to branch formed by *L. guilinensis, L. indica* “Lüzhao Hongdie,” and *L. indica. L. speciosa* alone was placed at the basal position, and showed a large divergence from the rest five *Lagerstroemia* taxa. The data presented here will facilitate the understanding of the evolutionary history of crape myrtles. These findings provide an informative and valuable genetic source of the *Lagerstroemia* germplasm resources for identifying species, elucidating taxonomy, and reconstructing the phylogeny of the *Lagerstroemia* genus.

## Author contributions

CX performed the experiments, analyzed the data, contributed reagents/materials/analysis tools, wrote the paper, reviewed drafts of the paper. WD conceived and designed the experiments, performed the experiments, analyzed the data, wrote the paper, prepared figures and/or tables, reviewed drafts of the paper. WL, YL, XX conceived and designed the experiments, contributed reagents/materials/analysis tools, wrote the paper, reviewed drafts of the paper. JS, KH contributed reagents/materials/analysis tools, reviewed drafts of the paper. XJ wrote the paper, reviewed drafts of the paper. ZS conceived and designed the experiments, performed the experiments, analyzed the data, contributed reagents/materials/analysis tools, wrote the paper, reviewed drafts of the paper.

## Funding

The study was financially supported by “Collection, Conservation, and Evaluation of Forest Tree Germplasm Resources” (LKZ201496-1-3) of Shandong Provincial Agricultural Elite Varieties Project, the joint projects No. 70009C1036 and 70009C1020, the National Natural Science Foundation of China (No. 30972412), and the National Forest Genetic Resources Platform (2005DKA21003).

### Conflict of interest statement

The authors declare that the research was conducted in the absence of any commercial or financial relationships that could be construed as a potential conflict of interest.

## References

[B1] APG III (2009). An update of the Angiosperm Phylogeny Group classification for the orders and families of flowering plants: APG III. Bot. J. Linnean Soc. 161, 105–121. 10.1111/j.1095-8339.2009.00996.x

[B2] Asaf S. Khan A. L. Khan A. R. Waqas M. Kang S. M. Khan M. A.. (2016). Complete chloroplast genome of *Nicotiana otophora* and its comparison with related species. Front. Plant Sci. 7:843. 10.3389/fpls.2016.00843 27379132 PMC4906380

[B3] Conant G. C. Wolfe K. H. (2008). GenomeVx: simple web-based creation of editable circular chromosome maps. Bioinformatics 24, 861–862. 10.1093/bioinformatics/btm598 18227121

[B4] Curci P. L. De Paola D. Danzi D. Vendramin G. G. Sonnante G. (2015). Complete chloroplast genome of the multifunctional crop globe artichoke and comparison with other Asteraceae. PLoS ONE 10:e0120589. 10.1371/journal.pone.0120589 25774672 PMC4361619

[B6] Dong W. P. Liu H. Xu C. Zuo Y. J. Chen Z. J. Zhou S. L. (2014). A chloroplast genomic strategy for designing taxon specific DNA mini-barcodes: a case study on ginsengs. BMC Genetics 15:138. 10.1186/s12863-014-0138-z 25526752 PMC4293818

[B5] Dong W. P. Liu J. Yu J. Wang L. Zhou S. L. (2012). Highly variable chloroplast markers for evaluating plant phylogeny at low taxonomic levels and for DNA barcoding. PLoS ONE 7:e35071. 10.1371/journal.pone.0035071 22511980 PMC3325284

[B7] Dong W. P. Xu C. Cheng T. Lin K. Zhou S. L. (2013). Sequencing angiosperm plastid genomes made easy: a complete set of universal primers and a case study on the phylogeny of Saxifragales. Genome Biol. Evol. 5, 989–997. 10.1093/gbe/evt063 23595020 PMC3673619

[B8] Dong W. P. Xu C. Li C. H. Sun J. H. Zuo Y. J. Shi S.. (2015). cf1, the most promising plastid DNA barcode of land plants. Sci. Rep. 5:8348. 10.1038/srep08348 25672218 PMC4325322

[B9] Dong W. P. Xu C. Li D. L. Jin X. B. Lu Q. Suo Z. L. (2016). Comparative analysis of the complete chloroplast genome sequences in psammophytic *Haloxylon* species (Amaranthaceae). Peer J. 4:e2699. 10.7717/peerj.2699 27867769 PMC5111891

[B10] Downie S. R. Jansen R. K. (2015). A comparative analysis of whole plastid genomes from the Apiales: expansion and contraction of the inverted repeat, mitochondrial to plastid transfer of DNA, and identification of highly divergent noncoding regions. Syst. Bot. 40, 336–351. 10.1600/036364415X686620

[B11] Frazer K. A. Pachter L. Poliakov A. Rubin E. M. Dubchak I. (2004). VISTA: computational tools for comparative genomics. Nucleic Acids Res. 32, W273–W279. 10.1093/nar/gkh458 15215394 PMC441596

[B12] Graham S. A. Hall J. Sytsma K. Shi S. (2005). Phylogenetic analysis of the Lythraceae based on four gene regions and morphology. Int. J. Plant Sci. 166, 995–1017. 10.1086/432631

[B13] Gu C. H. Tembrock L. R. Zhang D. Wu Z. Q. (2016b). Characterize the complete chloroplast genome of *Lagerstroemia floribunda* (Lythraceae), a narrow endemic crape myrtle native to Southeast Asia. Conserv. Genet. Resour. 10.1007/s12686-016-0628-6

[B14] Gu C. H. Tembrock L. R. Johnson N. G. Simmons M. P. Wu Z. (2016a). The complete plastid genome of *Lagerstroemia fauriei* and loss of *rpl2* intron from Lagerstroemia (Lythraceae). PLoS ONE 11:e0150752. 10.1371/journal.pone.0150752 26950701 PMC4780714

[B15] He D. Liu Y. Cai M. Pan H. T. Zhang Q. X. (2014). The first genetic linkage map of crape myrtle (Lagerstroemia) based on amplification fragment length polymorphisms and simple sequence repeats markers. Plant Breed. 133, 138–144. 10.1111/pbr.12100

[B16] Huang J. M. Hou B. X. Suo Z. L. (2013a). Study on the *Lagerstroemia indica* cultivars in Shaoyang city I. J. Agr. 3, 47–53. Available online at: http://www.caaj.org/cjas/ch/reader/view_abstract.aspx?

[B17] Huang J. M. Hou B. X. Suo Z. L. (2013b). Study on the *Lagerstroemia indica* cultivars in Shaoyang city II. J. Agr. 3, 35–41. Available online at: http://www.caaj.org/cjas/ch/reader/view_abstract.aspx?

[B18] Huang J. M. Hou B. X. Suo Z. L. (2013c). Study on the *Lagerstroemia indica* cultivars in Shaoyang city III. J. Agr. 3, 34–41. Available online at: http://www.caaj.org/cjas/ch/reader/view_abstract.aspx?

[B19] Huang Y. L. Shi S. H. (2002). Phylogenetics of Lythraceae sensu lato: a preliminary analysis based on chloroplast rbcL gene, psaA–ycf3 spacer, and nuclear rDNA internal transcribed spacer (ITS). Int. J. Plant Sci. 163, 215–225. 10.1086/338392

[B20] Jansen R. K. Cai Z. Q. Raubeson L. A. Daniell H. dePamphilis C. W. Leebens-Mack J.. (2007). Analysis of 81 genes from 64 plastid genomes resolves relationships in angiosperms and identifies genome scale evolutionary patterns. Proc. Natl. Acad. Sci. U.S.A. 104, 19369–19374. 10.1073/pnas.0709121104 18048330 PMC2148296

[B21] Katoh K. Standley D. M. (2013). MAFFT multiple sequence alignment software version 7: improvements in performance and usability. Mol. Biol. Evol. 30, 772–780. 10.1093/molbev/mst010 23329690 PMC3603318

[B22] Kaur S. Panesar P. S. Bera M. B. Kaur V. (2015). Simple sequence repeat markers in genetic divergence and marker-assisted selection of rice cultivars: A review. Crit. Rev. Food Sci. Nutr. 55, 41–49. 10.1080/10408398.2011.646363 24915404

[B23] Kim K. J. Lee H. L. (2005). Wide spread occurrence of small inversions in the chloroplast genomes of land plants. Mol. Cells 19, 104–113. Available online at: https://www.baidu.com/link?url=_noO3HXu9B7KSoCqEjSECCzJckjU3OwgkNNvQT3sPw7_wr-95HX5cri0P9UPrPePJpQMe69A_Isr-XWzxXa77jeYNdL0La5CPmpgck-_ZqGNiaUFEwPJkPNOoH4H1AFQ&wd=&eqid=9cda33380000a59b000000035873c1bd 15750347

[B24] Librado P. Rozas J. (2009). DnaSP v5: a software for comprehensive analysis of DNA polymorphism data. Bioinformatics 25, 1451–1452. 10.1093/bioinformatics/btp187 19346325

[B25] Moore M. J. Bell C. D. Soltis P. S. Soltis D. E. (2007). Using plastid genome-scale data to resolve enigmatic relationships among basal angiosperms. Proc. Natl. Acad. Sci. U.S.A. 104, 19363–19368. 10.1073/pnas.0708072104 18048334 PMC2148295

[B26] Morton B. R. Clegg M. T. (1995). Neighboring base composition is strongly correlated with base substitution bias in a region of the chloroplast genome. J. Mol. Evol. 41, 597–603. 10.1007/BF00175818 7490774

[B27] Morton B. R. Oberholzer V. M. Clegg M. T. (1997). The influence of specific neighboring bases on substitution bias in noncoding regions of the plant chloroplast genome. J. Mol. Evol. 45, 227–231. 10.1007/PL00006224 9302315

[B28] Pounders C. Rinehart T. Sakhanokho H. (2007). Evaluation of inter-specific hybrids between *Lagerstroemia indica* and *L*. speciosa. HortScience 42, 1317–1322. Available online at: http://hortsci.ashspublications.org/content/42/6/1317.full

[B29] Qin H. N. Shirley G. (2007). Lagerstroemia Linnaeus. Flora China 13, 277–281. Available online at: http://foc.eflora.cn/cncontent.aspx?TaxonId=117489

[B30] Raman G. Park S. (2015). Analysis of the complete chloroplast genome of a medicinal plant, *Dianthus superbus* var. longicalyncinus, from a comparative genomics perspective. PLoS ONE 10:e0141329. 10.1371/journal.pone.0141329 26513163 PMC4626046

[B31] Raman G. Park S. (2016). The complete chloroplast genome sequence of *Ampelopsis*: gene organization, comparative analysis, and phylogenetic relationships to other angiosperms. Front. Plant Sci. 7:341. 10.3389/fpls.2016.00341 27047519 PMC4800181

[B32] Rambaut A. (1996). Se-Al: Sequence Alignment Editor. Version 2.0. Oxford: University of Oxford, Department of Zoology.

[B33] Ronquist F. Teslenko M. van der Mark P. Ayres D. L. Darling A. Hohna S.. (2012). MrBayes 3.2: efficient Bayesian phylogenetic inference and model choice across a large model space. Syst. Biol. 61, 539–542. 10.1093/sysbio/sys029 22357727 PMC3329765

[B34] Song Y. Dong W. Liu B. Xu C. Yao X. Gao J.. (2015). Comparative analysis of complete chloroplast genome sequences of two tropical trees *Machilus yunnanensis* and *Machilus balansae* in the family Lauraceae. Front. Plant Sci. 6:662. 10.3389/fpls.2015.00662 26379689 PMC4548089

[B35] Stamatakis A. (2006). RAxML-VI-HPC: maximum likelihood-based phylogenetic analyses with thousands of taxa and mixed models. Bioinformatics 22, 2688–2690. 10.1093/bioinformatics/btl446 16928733

[B36] Su H. J. Hogenhout S. A. Al-Sadi A. M. Kuo C. H. (2014). Complete chloroplast genome sequence of omani lime (*Citrus aurantiifolia*) and comparative analysis within the Rosids. PLoS ONE 9:e113049. 10.1371/journal.pone.0113049 25398081 PMC4232571

[B37] Suo Z. L. Chen L. N. Pei D. Jin X. B. Zhang H. J. (2015). A new nuclear DNA marker from ubiquitin ligase gene region for genetic diversity detection of walnut germplasm resources. Biotechnol. Rep. 5, 40–45. 10.1016/j.btre.2014.11.003 PMC5466192 28626681

[B38] Suo Z. L. Li W. Y. Jin X. B. Zhang H. J. (2016). A new nuclear DNA marker revealing both microsatellite variations and single nucleotide polymorphic loci: a case study on classification of cultivars in *Lagerstroemia indica* L. J. Microb. Biochem. Technol. 8, 266–271. 10.4172/1948-5948.1000296

[B39] Suo Z. L. Zhang C. H. Zheng Y. Q. He L. X. Jin X. B. Hou B. X.. (2012). Revealing genetic diversity of tree peonies at micro-evolution level with hyper-variable chloroplast markers and floral traits. Plant Cell Rep. 31, 2199–2213. 10.1007/s00299-012-1330-0 22961193

[B40] Swofford D. L. (2003). PAUP^*^. Phylogenetic Analysis Using Parsimony (^*^and Other Methods). Version4b10. Sunderland, MA: Sinauer.

[B41] Tamura K. Stecher G. Peterson D. Filipski A. Kumar S. (2013). MEGA6: molecular evolutionary genetics analysis version 6.0. Mol. Biol. Evol. 30, 2725–2729. 10.1093/molbev/mst197 24132122 PMC3840312

[B42] Thiel T. Michalek W. Varshney R. Graner A. (2003). Exploiting EST databases for the development and characterization of gene-derived SSR-markers in barley (*Hordeum vulgare* L.). Theor. Appl. Genet. 106, 411–422. 10.1007/s00122-002-1031-0 12589540

[B43] Wang X. Wadlb P. A. Pounders C. Trigiano R. N. Cabrera R. I. Scheffler B. E. (2011). Evaluation of genetic diversity and pedigree within crapemyrtle cultivars using simple sequence repeat markers. J. Amer. Soc. Hort. Sci. 136, 116–128. Available online at: http://journal.ashspublications.org/content/136/2/116.full

[B44] Wyman S. K. Jansen R. K. Boore J. L. (2004). Automatic annotation of organellar genomes with DOGMA. Bioinformatics 20, 3252–3255. 10.1093/bioinformatics/bth352 15180927

[B45] Xiang X. G. Zhang J. B. Lu A. M. Li R. Q. (2011). Molecular identification on species in Juglandaceae: a tiered method. J. Syst. Evol. 49, 252–260. 10.1111/j.1759-6831.2011.00116.x

[B46] Yang Y. Zhou T. Duan D. Yang J. Feng L. Zhao G. (2016). Comparative analysis of the complete chloroplast genomes of five Quercus species. Front. Plant Sci. 7:959. 10.3389/fpls.2016.00959 27446185 PMC4923075

[B47] Yang Z. H. (2007). PAML 4: Phylogenetic analysis by maximum likelihood. Mol. Biol. Evol. 24, 1586–1591. 10.1093/molbev/msm088 17483113

[B48] Zhang Y. Du L. Liu A. Chen J. Wu L. Hu W.. (2016). The complete chloroplast genome sequences of five *Epimedium* species: lights into phylogenetic and taxonomic analyses. Front. Plant Sci. 7:306. 10.3389/fpls.2016.00306 27014326 PMC4791396

