# Supplementary material for: Comparative Analysis of Six Lagerstroemia Complete Chloroplast Genomes
Source: Front Plant Sci. 2017 Jan 19;8:15. doi: 10.3389/fpls.2017.00015 (PMC5243828; doi:10.3389/fpls.2017.00015)
Supplement: Supplementary file 2 [file Table2.DOCX]

**TABLE S2 | Features of SSRs in each of the six *Lagerstroemia* chloroplast genomes.**

| **Taxa** | **SSR type** | **SSR** | **Size** | **Start** | **End** |  | **Locus** | **Location** | **Region** |
| --- | --- | --- | --- | --- | --- | --- | --- | --- | --- |
| *L. fauriei* | p1 | **(T) ×10** | 10 | 1554 | 1563 |  | *trn*K-*mat*K | intergenic | LSC |
| *L. fauriei* | p1 | **(G) ×13** | 13 | 4688 | 4700 |  | *mat*K-*rps*16 | intergenic | LSC |
| *L. fauriei* | p1 | **(A) ×14** | 14 | 6836 | 6849 |  | *trn*Q-*psb*K | intergenic | LSC |
| *L. fauriei* | p1 | **(A) ×10** | 10 | 11860 | 11869 |  | *atp*F intron | intron | LSC |
| *L. fauriei* | p1 | **(T) ×13** | 13 | 12528 | 12540 |  | *atp*F-atpH | intergenic | LSC |
| *L. fauriei* | p1 | **(T) ×10** | 10 | 25460 | 25469 |  | *rpo*B | gene | LSC |
| *L. fauriei* | p1 | **(T) ×10** | 10 | 28060 | 28069 |  | *pet*N-*psb*M | intergenic | LSC |
| *L. fauriei* | p1 | **(T) × 11** | 11 | 49396 | 49406 |  | *ndh*C-*trn*V | intergenic | LSC |
| *L. fauriei* | p1 | **(T) ×11** | 11 | 50233 | 50243 |  | *ndh*C-*trn*V | intergenic | LSC |
| *L. fauriei* | p1 | **(T) ×10** | 10 | 56836 | 56845 |  | *rbc*L-*acc*D | intergenic | LSC |
| *L. fauriei* | p1 | **(A) ×10** | 10 | 60779 | 60788 |  | *ycf*4-*cem*A | intergenic | LSC |
| *L. fauriei* | p1 | **(A) ×10** | 10 | 65089 | 65098 |  | *psb*E-*pet*L | intergenic | LSC |
| *L. fauriei* | p1 | **(T) × 10** | 10 | 66410 | 66419 |  | *trn*P-*psa*J | intergenic | LSC |
| *L. fauriei* | p1 | **(T) ×10** | 10 | 67386 | 67395 |  | *psa*J-*rpl*33 | intergenic | LSC |
| *L. fauriei* | p1 | **(A) × 11** | 11 | 68275 | 68285 |  | *rps*18-*rp*l20 | intergenic | LSC |
| *L. fauriei* | p1 | **(A) ×10** | 10 | 70374 | 70383 |  | *clp*P intron 1 | intron | LSC |
| *L. fauriei* | p1 | **(T) ×11** | 11 | 75105 | 75115 |  | *pet*B intron | intron | LSC |
| *L. fauriei* | p1 | **(T) ×10** | 10 | 78874 | 78883 |  | *rpo*A-*rps*11 | intergenic | LSC |
| *L. fauriei* | p1 | **(T) ×11** | 11 | 94727 | 94737 |  | *ndh*B intron | intron | IRa |
| *L. fauriei* | p1 | **(A) ×10** | 10 | 106529 | 106538 |  | *rrn*5-*trn*R | intergenic | IRa |
| *L. fauriei* | p1 | **(T) ×10** | 10 | 110447 | 110456 |  | *ndh*F | gene | SSC |
| *L. fauriei* | p1 | **(A) ×11** | 11 | 112022 | 112032 |  | *ndh*F-*rpl*32 | intergenic | SSC |
| *L. fauriei* | p1 | **(T) ×11** | 11 | 112058 | 112068 |  | *ndh*F-*rpl*32 | intergenic | SSC |
| *L. fauriei* | p1 | **(A) ×10** | 10 | 120185 | 120194 |  | *ndh*A intron | intron | SSC |
| *L. fauriei* | p1 | **(T) ×11** | 11 | 124224 | 124234 |  | *ycf*1 | gene | SSC |
| *L. fauriei* | p1 | **(T) ×10** | 10 | 124788 | 124797 |  | *ycf*1 | gene | SSC |
| *L. fauriei* | p1 | **(T) ×10** | 10 | 129821 | 129830 |  | *trn*R-*rrn*5 | intergenic | IRb |
| *L. fauriei* | p1 | **(A) ×11** | 11 | 141622 | 141632 |  | *ndh*B intron | intron | IRb |
| *L. guilinensis* | p1 | **(T) × 10** | 10 | 1554 | 1563 |  | *trn*K-*mat*K | intergenic | LSC |
| *L. guilinensis* | p1 | **(G) ×13** | 13 | 4685 | 4697 |  | *mat*K-*rps*16 | intergenic | LSC |
| *L. guilinensis* | p1 | **(T) ×11** | 11 | 7250 | 7260 |  | *psb*K-*psb*I | intergenic | LSC |
| *L. guilinensis* | p1 | **(T) ×11** | 11 | 11070 | 11080 |  | *atp*A-*atp*F | intergenic | LSC |
| *L. guilinensis* | p1 | **(T) × 11** | 11 | 12618 | 12628 |  | *atp*F-*atp*H | intergenic | LSC |
| *L. guilinensis* | p1 | **(T) ×10** | 10 | 25548 | 25557 |  | *rpo*B | gene | LSC |
| *L. guilinensis* | p1 | **(T) × 12** | 12 | 50327 | 50338 |  | *ndh*C-*trn*V | intergenic | LSC |
| *L. guilinensis* | p1 | **(A) × 10** | 10 | 53998 | 54007 |  | *atp*B-*rbc*L | intergenic | LSC |
| *L. guilinensis* | p1 | **(A) × 10** | 10 | 60663 | 60672 |  | *ycf*4-*cem*A | intergenic | LSC |
| *L. guilinensis* | p1 | **(T) × 10** | 10 | 64472 | 64481 |  | *psb*E-*pet*L | intergenic | LSC |
| *L. guilinensis* | p1 | **(A) × 10** | 10 | 64973 | 64982 |  | *psb*E-*pet*L | intergenic | LSC |
| *L. guilinensis* | p1 | **(T) × 10** | 10 | 66294 | 66303 |  | *trn*P-*psa*J | intergenic | LSC |
| *L. guilinensis* | p1 | **(A) × 10** | 10 | 68166 | 68175 |  | *rps*18-*rpl*20 | intergenic | LSC |
| *L. guilinensis* | p1 | **(T) × 12** | 12 | 74993 | 75004 |  | *pet*B intron | intron | LSC |
| *L. guilinensis* | p1 | **(T) × 10** | 10 | 78763 | 78772 |  | *rpo*A-*rps*11 | intergenic | LSC |
| *L. guilinensis* | p1 | **(T) × 11** | 11 | 94618 | 94628 |  | *ndh*B intron | intron | IRa |
| *L. guilinensis* | p1 | **(A) × 10** | 10 | 106305 | 106314 |  | *rrn*5-*trn*R | intergenic | IRa |
| *L. guilinensis* | p1 | **(T) × 10** | 10 | 110223 | 110232 |  | *ndh*F | gene | SSC |
| *L. guilinensis* | p1 | **(A) × 10** | 10 | 111798 | 111807 |  | *ndh*F-*rpl*32 | intergenic | SSC |
| *L. guilinensis* | p1 | **(T) × 10** | 10 | 117937 | 117946 |  | *ndh*G-*ndh*I | intergenic | SSC |
| *L. guilinensis* | p1 | **(T) × 11** | 11 | 123969 | 123979 |  | *ycf*1 | gene | SSC |
| *L. guilinensis* | p1 | **(T) × 10** | 10 | 124533 | 124542 |  | *ycf*1 | gene | SSC |
| *L. guilinensis* | p1 | **(T) × 10** | 10 | 129572 | 129581 |  | *trn*R-*rrn*5 | intergenic | IRb |
| *L. guilinensis* | p1 | **(A) × 11** | 11 | 141377 | 141387 |  | *ndh*B intron | intron | IRb |
| *L. indica* | p1 | **(T) × 11** | 11 | 1555 | 1565 |  | *trn*K-*mat*K | intergenic | LSC |
| *L. indica* | p1 | **(T) × 10** | 10 | 7248 | 7257 |  | *psb*K-*psb*I | intergenic | LSC |
| *L. indica* | p1 | **(T) × 10** | 10 | 11067 | 11076 |  | *atp*A-*atp*F | intergenic | LSC |
| *L. indica* | p1 | **(A) × 10** | 10 | 11947 | 11956 |  | *atp*F intron | intron | LSC |
| *L. indica* | p1 | **(T) × 10** | 10 | 12615 | 12624 |  | *atp*F-*atp*H | intergenic | LSC |
| *L. indica* | p1 | **(T) × 10** | 10 | 25544 | 25553 |  | *rpo*B | gene | LSC |
| *L. indica* | p1 | **(T) × 10** | 10 | 29052 | 29061 |  | *psb*M-*trn*D | intergenic | LSC |
| *L. indica* | p1 | **(T) ×10** | 10 | 50323 | 50332 |  | *ndh*C-*trn*V | intergenic | LSC |
| *L. indica* | p1 | **(A) × 10** | 10 | 65212 | 65221 |  | *psb*E-*pet*L | intergenic | LSC |
| *L. indica* | p1 | **(A) × 10** | 10 | 68404 | 68413 |  | *rps*18-*rpl*20 | intergenic | LSC |
| *L. indica* | p1 | **(T) × 10** | 10 | 75231 | 75240 |  | *pet*B intron | intron | LSC |
| *L. indica* | p1 | **(T) × 10** | 10 | 94853 | 94862 |  | *ndh*B intron | intron | IRa |
| *L. indica* | p1 | **(T) × 10** | 10 | 110403 | 110412 |  | *ndh*F | gene | SSC |
| *L. indica* | p1 | **(A) × 10** | 10 | 111977 | 111986 |  | *ndh*F-*rpl*32 | intergenic | SSC |
| *L. indica* | p1 | **(T) × 10** | 10 | 118125 | 118134 |  | *ndh*G-*ndh*I | intergenic | SSC |
| *L. indica* | p1 | **(A)× 10** | 10 | 120117 | 120126 |  | *ndh*A intron | intron | SSC |
| *L. indica* | p1 | **(T) ×11** | 11 | 124156 | 124166 |  | *ycf*1 | gene | SSC |
| *L. indica* | p1 | **(A) ×10** | 10 | 141390 | 141399 |  | *ndh*B intron | intron | IRb |
| *L. indica* ‘Lüzhao Hongdie’ | p1 | **(T) ×10** | 10 | 1555 | 1564 |  | *trn*K-*mat*K | intergenic | LSC |
| *L. indica* ‘Lüzhao Hongdie’ | p1 | **(G) ×14** | 14 | 4685 | 4698 |  | *trn*K-*rps*16 | intergenic | LSC |
| *L. indica* ‘Lüzhao Hongdie’ | p1 | **(T) ×10** | 10 | 7253 | 7262 |  | *psb*K-*psb*I | intergenic | LSC |
| *L. indica* ‘Lüzhao Hongdie’ | p1 | **(T) ×10** | 10 | 11072 | 11081 |  | *atp*A-*atp*F | intergenic | LSC |
| *L. indica* ‘Lüzhao Hongdie’ | p1 | **(A) ×10** | 10 | 11952 | 11961 |  | *atp*F intron | intron | LSC |
| *L. indica* ‘Lüzhao Hongdie’ | p1 | **(T) ×11** | 11 | 12620 | 12630 |  | *atp*F-*atp*H | intergenic | LSC |
| *L. indica* ‘Lüzhao Hongdie’ | p1 | **(T) ×10** | 10 | 25550 | 25559 |  | *rpo*B | gene | LSC |
| *L. indica* ‘Lüzhao Hongdie’ | p1 | **(T) ×10** | 10 | 29058 | 29067 |  | *psb*M-*trn*D | intergenic | LSC |
| *L. indica* ‘Lüzhao Hongdie’ | p1 | **(T) × 11** | 11 | 50329 | 50339 |  | *ndh*C-*trn*V | intergenic | LSC |
| *L. indica* ‘Lüzhao Hongdie’ | p1 | **(A) ×10** | 10 | 54211 | 54220 |  | *atp*B-*rbc*L | intergenic | LSC |
| *L. indica* ‘Lüzhao Hongdie’ | p1 | **(T) ×10** | 10 | 64719 | 64728 |  | *psb*E-*pet*L | intergenic | LSC |
| *L. indica* ‘Lüzhao Hongdie’ | p1 | **(A) ×10** | 10 | 65220 | 65229 |  | *psb*E-*pet*L | intergenic | LSC |
| *L. indica* ‘Lüzhao Hongdie’ | p1 | **(T) ×10** | 10 | 66541 | 66550 |  | *trn*P-*psa*J | intergenic | LSC |
| *L. indica* ‘Lüzhao Hongdie’ | p1 | **(A) ×10** | 10 | 68413 | 68422 |  | *rps*18-*rpl*20 | intergenic | LSC |
| *L. indica* ‘Lüzhao Hongdie’ | p1 | **(A) ×10** | 10 | 71438 | 71447 |  | *clp*P intron 1 | intron | LSC |
| *L. indica* ‘Lüzhao Hongdie’ | p1 | **(A) ×10** | 10 | 71767 | 71776 |  | *clp*P intron 1 | intron | LSC |
| *L. indica* ‘Lüzhao Hongdie’ | p1 | **(T) ×10** | 10 | 75242 | 75251 |  | *pet*B intron | intron | LSC |
| *L. indica* ‘Lüzhao Hongdie’ | p1 | **(T) ×10** | 10 | 79010 | 79019 |  | *rpo*A-*rps*11 | intergenic | LSC |
| *L. indica* ‘Lüzhao Hongdie’ | p1 | **(T) × 11** | 11 | 94869 | 94879 |  | *ndh*B intron | intron | IRa |
| *L. indica* ‘Lüzhao Hongdie’ | p1 | **(A) × 10** | 10 | 106504 | 106513 |  | *rrn*5-*trn*R | intergenic | IRa |
| *L. indica* ‘Lüzhao Hongdie’ | p1 | **(T) ×10** | 10 | 110422 | 110431 |  | *ndh*F | gene | SSC |
| *L. indica* ‘Lüzhao Hongdie’ | p1 | **(A) × 10** | 10 | 111996 | 112005 |  | *ndh*F-*rpl*32 | intergenic | SSC |
| *L. indica* ‘Lüzhao Hongdie’ | p1 | **(T) × 10** | 10 | 112636 | 112645 |  | *ndh*F-*rpl*32 | intergenic | SSC |
| *L. indica* ‘Lüzhao Hongdie’ | p1 | **(C) × 10** | 10 | 113644 | 113653 |  | *trn*L-*ccs*A | intergenic | SSC |
| *L. indica* ‘Lüzhao Hongdie’ | p1 | **(T) ×10** | 10 | 118147 | 118156 |  | *ndh*G-*ndh*I | intergenic | SSC |
| *L. indica* ‘Lüzhao Hongdie’ | p1 | **(T) ×11** | 11 | 124178 | 124188 |  | *ycf*1 | gene | SSC |
| *L. indica* ‘Lüzhao Hongdie’ | p1 | **(T) ×10** | 10 | 124742 | 124751 |  | *ycf*1 | gene | SSC |
| *L. indica* ‘Lüzhao Hongdie’ | p1 | **(T) × 10** | 10 | 129781 | 129790 |  | *trn*R-*rrn*5 | intergenic | IRb |
| *L. indica* ‘Lüzhao Hongdie’ | p1 | **(A) × 11** | 11 | 141415 | 141425 |  | *ndh*B intron | intron | IRb |
| *L. speciosa* | p1 | **(A) ×10** | 10 | 4089 | 4098 |  | *trn*K-*rps*16 | intergenic | LSC |
| *L. speciosa* | p1 | **(A) × 10** | 10 | 4235 | 4244 |  | *trn*K-*rps*16 | intergenic | LSC |
| *L. speciosa* | p1 | **(G) × 10** | 10 | 4652 | 4661 |  | *trn*K-*rps*16 | intergenic | LSC |
| *L. speciosa* | p1 | **(T) × 10** | 10 | 6277 | 6286 |  | *rps*16-*trn*Q | intergenic | LSC |
| *L. speciosa* | p1 | **(T) × 10** | 10 | 10975 | 10984 |  | *atp*A-*atp*F | intergenic | LSC |
| *L. speciosa* | p1 | **(T) × 11** | 11 | 12525 | 12535 |  | *atp*F-*atp*H | intergenic | LSC |
| *L. speciosa* | p1 | **(T) × 13** | 13 | 13747 | 13759 |  | *atp*H-*atp*I | intergenic | LSC |
| *L. speciosa* | p1 | **(A) × 10** | 10 | 15710 | 15719 |  | *rps*2-*rpo*C2 | intergenic | LSC |
| *L. speciosa* | p1 | **(T) × 10** | 10 | 25451 | 25460 |  | *rpo*B | gene | LSC |
| *L. speciosa* | p1 | **(T) × 10** | 10 | 27290 | 27299 |  | *trn*C-*pet*N | intergenic | LSC |
| *L. speciosa* | p1 | **(T) × 11** | 11 | 28056 | 28066 |  | *pet*N-*psb*M | intergenic | LSC |
| *L. speciosa* | p1 | **(A) ×10** | 10 | 31295 | 31304 |  | *trn*T-*psb*D | intergenic | LSC |
| *L. speciosa* | p1 | **(A) ×14** | 14 | 35979 | 35992 |  | *psb*Z-*trn*G | intergenic | LSC |
| *L. speciosa* | p1 | **(T) ×10** | 10 | 50614 | 50623 |  | *ndh*C-*trn*V | intergenic | LSC |
| *L. speciosa* | p1 | **(T) ×10** | 10 | 64946 | 64955 |  | *psb*E-*pet*L | intergenic | LSC |
| *L. speciosa* | p1 | **(A) ×10** | 10 | 65447 | 65456 |  | *psb*E-*pet*L | intergenic | LSC |
| *L. speciosa* | p1 | **(T) ×10** | 10 | 66773 | 66782 |  | *trn*P-*psa*J | intergenic | LSC |
| *L. speciosa* | p1 | **(T) ×10** | 10 | 75357 | 75366 |  | *pet*B intron | intron | LSC |
| *L. speciosa* | p1 | **(C) ×12** | 12 | 75371 | 75382 |  | *pet*B intron | intron | LSC |
| *L. speciosa* | p1 | **(T) × 10** | 10 | 81898 | 81907 |  | *rpl*16 intron | intron | LSC |
| *L. speciosa* | p1 | **(T) × 11** | 11 | 94998 | 95008 |  | *ndh*B intron | intron | IRa |
| *L. speciosa* | p1 | **(T) × 10** | 10 | 117717 | 117726 |  | *ndh*E-*ndh*G | intergenic | SSC |
| *L. speciosa* | p1 | **(T) × 10** | 10 | 124924 | 124933 |  | *ycf*1 | gene | SSC |
| *L. speciosa* | p1 | **(A) × 11** | 11 | 141712 | 141722 |  | *ndh*B intron | intron | IRb |
| *L. subcostata* | p1 | **(A) × 11** | 11 | 4076 | 4086 |  | *trn*K-*rps*16 | intergenic | LSC |
| *L. subcostata* | p1 | **(G) × 10** | 10 | 4689 | 4698 |  | *trn*K-*rps*16 | intergenic | LSC |
| *L. subcostata* | p1 | **(A) ×15** | 15 | 6834 | 6848 |  | *trn*Q-*psb*K | intergenic | LSC |
| *L. subcostata* | p1 | **(T) × 10** | 10 | 7258 | 7267 |  | *psb*K-*psb*I | intergenic | LSC |
| *L. subcostata* | p1 | **(T) ×14** | 14 | 12520 | 12533 |  | *atp*F-*atp*H | intergenic | LSC |
| *L. subcostata* | p1 | **(T) × 10** | 10 | 25438 | 25447 |  | *rpo*B | gene | LSC |
| *L. subcostata* | p1 | **(T) × 10** | 10 | 49361 | 49370 |  | *ndh*K-*ndh*C | intergenic | LSC |
| *L. subcostata* | p1 | **(T) × 11** | 11 | 50196 | 50206 |  | ndhC-trnV | intergenic | LSC |
| *L. subcostata* | p1 | **(A) × 10** | 10 | 60749 | 60758 |  | *ycf*4-*cem*A | intergenic | LSC |
| *L. subcostata* | p1 | **(T) × 10** | 10 | 66379 | 66388 |  | *trn*P-*psa*J | intergenic | LSC |
| *L. subcostata* | p1 | **(T) × 10** | 10 | 67355 | 67364 |  | *psa*J-*rpl*33 | intergenic | LSC |
| *L. subcostata* | p1 | **(A) ×10** | 10 | 71594 | 71603 |  | *clp*P intron 1 | intron | LSC |
| *L. subcostata* | p1 | **(T) ×12** | 12 | 75072 | 75083 |  | *pet*B intron | intron | LSC |
| *L. subcostata* | p1 | **(T) ×10** | 10 | 78842 | 78851 |  | *rpo*A-*rps*11 | intergenic | LSC |
| *L. subcostata* | p1 | **(T) × 10** | 10 | 81595 | 81604 |  | *rpl*16 intron | intron | LSC |
| *L. subcostata* | p1 | **(T) × 11** | 11 | 94697 | 94707 |  | *ndh*B intron | intron | IRa |
| *L. subcostata* | p1 | **(A) × 10** | 10 | 106332 | 106341 |  | *rrn*5-*trn*R | intergenic | IRa |
| *L. subcostata* | p1 | **(T) × 10** | 10 | 110250 | 110259 |  | *ndh*F | gene | SSC |
| *L. subcostata* | p1 | **(T) × 10** | 10 | 111859 | 111868 |  | *ndh*F-*rpl*32 | intergenic | SSC |
| *L. subcostata* | p1 | **(T) × 10** | 10 | 117966 | 117975 |  | *ndh*G-*ndh*I | intergenic | SSC |
| *L. subcostata* | p1 | **(A) × 10** | 10 | 119957 | 119966 |  | *ndh*A intron | intron | SSC |
| *L. subcostata* | p1 | **(T) × 11** | 11 | 124002 | 124012 |  | *ycf*1 | gene | SSC |
| *L. subcostata* | p1 | **(T) × 10** | 10 | 124566 | 124575 |  | *ycf*1 | gene | SSC |
| *L. subcostata* | p1 | **(T) × 10** | 10 | 129599 | 129608 |  | *trn*R-*rrn*5 | intergenic | IRb |
| *L. subcostata* | p1 | **(A) ×11** | 11 | 141233 | 141243 |  | *ndh*B intron | intron | IRb |
| *L. fauriei* | p2 | **(AT) × 5** | 10 | 19152 | 19161 |  | *rpo*C2 | gene | LSC |
| *L. fauriei* | p2 | **(TA) × 5** | 10 | 59041 | 59050 |  | *acc*D-*psa*I | intergenic | LSC |
| *L. fauriei* | p2 | **(TC) × 5** | 10 | 60922 | 60931 |  | *cem*A | gene | LSC |
| *L. fauriei* | p2 | **(AT) × 5** | 10 | 119848 | 119857 |  | *ndh*A intron | intron | SSC |
| *L. guilinensis* | p2 | **(AT) × 5** | 10 | 19241 | 19250 |  | *rpo*C2 | gene | LSC |
| *L. guilinensis* | p2 | **(AT) × 5** | 10 | 41563 | 41572 |  | *psa*A-*ycf*3 | intergenic | LSC |
| *L. guilinensis* | p2 | **(TA) × 5** | 10 | 58925 | 58934 |  | *acc*D-*psa*I | intergenic | LSC |
| *L. guilinensis* | p2 | **(TC) × 5** | 10 | 60806 | 60815 |  | *cem*A | gene | LSC |
| *L. guilinensis* | p2 | **(AT) × 5** | 10 | 119591 | 119600 |  | *ndh*A intron | intron | SSC |
| *L. indica* | p2 | **(AT) × 5** | 10 | 19237 | 19246 |  | *rpo*C2 | gene | LSC |
| *L. indica* | p2 | **(AT) ×5** | 10 | 41560 | 41569 |  | *psa*A-*ycf*3 | intergenic | LSC |
| *L. indica* | p2 | **(AT) ×5** | 10 | 44239 | 44248 |  | *ycf*3-*trn*S | intergenic | LSC |
| *L. indica* | p2 | **(TA) ×5** | 10 | 59166 | 59175 |  | *acc*D-*psa*I | intergenic | LSC |
| *L. indica* | p2 | **(TC) ×5** | 10 | 61046 | 61055 |  | *cem*A | gene | LSC |
| *L. indica* | p2 | **(AT) × 5** | 10 | 119779 | 119788 |  | *ndh*A intron | intron | SSC |
| *L. indica* ‘Lüzhao Hongdie’ | p2 | **(AT) × 5** | 10 | 19243 | 19252 |  | *rpo*C2 | gene | LSC |
| *L. indica* ‘Lüzhao Hongdie’ | p2 | **(AT) × 5** | 10 | 41566 | 41575 |  | *psa*A-*ycf*3 | intergenic | LSC |
| *L. indica* ‘Lüzhao Hongdie’ | p2 | **(TA) ×5** | 10 | 59174 | 59183 |  | *acc*D-*psa*I | intergenic | LSC |
| *L. indica* ‘Lüzhao Hongdie’ | p2 | **(TC) ×5** | 10 | 61054 | 61063 |  | *cem*A | gene | LSC |
| *L. indica* ‘Lüzhao Hongdie’ | p2 | **(AT) × 5** | 10 | 119801 | 119810 |  | *ndh*F-*rpl*32 | intergenic | SSC |
| *L. speciosa* | p2 | **(AT) ×5** | 10 | 19138 | 19147 |  | *rpo*C2 | gene | LSC |
| *L. speciosa* | p2 | **(AT) ×5** | 10 | 28647 | 28656 |  | *pet*N-*psb*M | intergenic | LSC |
| *L. speciosa* | p2 | **(TA) ×5** | 10 | 59397 | 59406 |  | *acc*D-*psa*I | intergenic | LSC |
| *L. speciosa* | p2 | **(TC) × 5** | 10 | 61287 | 61296 |  | *cem*A | gene | LSC |
| *L. speciosa* | p2 | **(TA) × 5** | 10 | 118556 | 118565 |  | *ndh*G-*ndh*I | intergenic | SSC |
| *L. speciosa* | p2 | **(AT) ×6** | 12 | 119992 | 120003 |  | *ndh*A intron | intron | SSC |
| *L. subcostata* | p2 | **(AT) ×5** | 10 | 19130 | 19139 |  | *rpo*C2 | gene | LSC |
| *L. subcostata* | p2 | **(TA) ×5** | 10 | 59011 | 59020 |  | *acc*D-*psa*I | intergenic | LSC |
| *L. subcostata* | p2 | **(TC) × 5** | 10 | 60892 | 60901 |  | *cem*A | gene | LSC |
| *L. subcostata* | p2 | **(AT) ×5** | 10 | 119620 | 119629 |  | *ndh*A intron | intron | SSC |
| *L. fauriei* | p3 | **(AAT) × 4** | 12 | 43521 | 43532 |  | *ycf*3 intron 2 | intron | LSC |
| *L. fauriei* | p3 | **(ATA) × 4** | 12 | 54051 | 54062 |  | *atp*B-*rbc*L | intergenic | LSC |
| *L. fauriei* | p3 | **(ATT) × 4** | 12 | 59085 | 59096 |  | *acc*D-*psa*I | intergenic | LSC |
| *L. fauriei* | p3 | **(AGA) ×4** | 12 | 87920 | 87931 |  | *ycf*2 | gene | IRa |
| *L. fauriei* | p3 | **(TTA) × 5** | 15 | 112145 | 112159 |  | *ndh*F-*rpl*32 | intergenic | SSC |
| *L. fauriei* | p3 | **(ATT) × 4** | 12 | 113522 | 113533 |  | *rpl*32-*trn*L | intergenic | SSC |
| *L. fauriei* | p3 | **(TTC) × 4** | 12 | 148427 | 148438 |  | *ycf*2 | gene | IRb |
| *L. guilinensis* | p3 | **(AAT) × 4** | 12 | 43616 | 43627 |  | *ycf*3 intron 2 | intron | LSC |
| *L. guilinensis* | p3 | **(ATA) × 4** | 12 | 53942 | 53953 |  | *atp*B-*rbc*L | intergenic | LSC |
| *L. guilinensis* | p3 | **(ATT) × 4** | 12 | 58969 | 58980 |  | *acc*D-*psa*I | intergenic | LSC |
| *L. guilinensis* | p3 | **(AGA) × 4** | 12 | 87811 | 87822 |  | *ycf*2 | gene | IRa |
| *L. guilinensis* | p3 | **(TTA) × 5** | 15 | 111917 | 111931 |  | *ndh*F-*rpl*32 | intergenic | SSC |
| *L. guilinensis* | p3 | **(ATT) × 4** | 12 | 113258 | 113269 |  | *rpl*32*-trn*L | intergenic | SSC |
| *L. guilinensis* | p3 | **(TTC) ×4** | 12 | 148182 | 148193 |  | *ycf*2 | gene | IRb |
| *L. indica* | p3 | **(AAT) × 4** | 12 | 43613 | 43624 |  | *ycf*3 intron 2 | intron | LSC |
| *L. indica* | p3 | **(ATA) × 4** | 12 | 54148 | 54159 |  | *atp*B-*rbc*L | intergenic | LSC |
| *L. indica* | p3 | **(ATT) × 4** | 12 | 59210 | 59221 |  | *acc*D-*psa*I | intergenic | LSC |
| *L. indica* | p3 | **(AGA) × 4** | 12 | 88046 | 88057 |  | *ycf*2 | gene | IRa |
| *L. indica* | p3 | **(TTA) × 5** | 15 | 112096 | 112110 |  | *ndh*F-*rpl*32 | intergenic | SSC |
| *L. indica* | p3 | **(ATT) × 4** | 12 | 113445 | 113456 |  | *rpl*32-*trn*L | intergenic | SSC |
| *L. indica* | p3 | **(TTC) × 4** | 12 | 148194 | 148205 |  | *ycf*2 | gene | IRb |
| *L. indica* ‘Lüzhao Hongdie’ | p3 | **(AAT) × 4** | 12 | 43619 | 43630 |  | *ycf*3 intron 2 | intron | LSC |
| *L. indica* ‘Lüzhao Hongdie’ | p3 | **(ATA) × 4** | 12 | 54155 | 54166 |  | *atp*B-*rbc*L | intergenic | LSC |
| *L. indica* ‘Lüzhao Hongdie’ | p3 | **(ATT) × 4** | 12 | 59218 | 59229 |  | *acc*D-*psa*I | intergenic | LSC |
| *L. indica* ‘Lüzhao Hongdie’ | p3 | **(AGA) × 4** | 12 | 88062 | 88073 |  | *ycf*2 | gene | IRa |
| *L. indica* ‘Lüzhao Hongdie’ | p3 | **(TTA) × 5** | 15 | 112115 | 112129 |  | *ndh*F-*rpl*32 | intergenic | SSC |
| *L. indica* ‘Lüzhao Hongdie’ | p3 | **(ATT) × 4** | 12 | 113464 | 113475 |  | *rpl*32-*trn*L | intergenic | SSC |
| *L. indica* ‘Lüzhao Hongdie’ | p3 | **(TTC) × 4** | 12 | 148220 | 148231 |  | *ycf*2 | gene | IRb |
| *L. speciosa* | p3 | **(TTA) × 4** | 12 | 12446 | 12457 |  | *atp*F-*atp*H | intergenic | LSC |
| *L. speciosa* | p3 | **(AAT) × 4** | 12 | 43904 | 43915 |  | *ycf*3 intron 2 | intron | LSC |
| *L. speciosa* | p3 | **(ATA) × 4** | 12 | 54399 | 54410 |  | *atp*B-*rbc*L | intergenic | LSC |
| *L. speciosa* | p3 | **(ATT) ×4** | 12 | 59441 | 59452 |  | *acc*D-*psa*I | intergenic | LSC |
| *L. speciosa* | p3 | **(AGA) ×4** | 12 | 88173 | 88184 |  | *ycf*2 | gene | IRa |
| *L. speciosa* | p3 | **(ATT) × 4** | 12 | 113696 | 113707 |  | *rpl*32-*trn*L | intergenic | SSC |
| *L. speciosa* | p3 | **(TTC) × 4** | 12 | 148535 | 148546 |  | *ycf*2 | gene | IRb |
| *L. subcostata* | p3 | **(AAT) × 4** | 12 | 43486 | 43497 |  | *ycf*3 intron 2 | intron | LSC |
| *L. subcostata* | p3 | **(ATA) × 4** | 12 | 54022 | 54033 |  | *atp*B-*rbc*L | intergenic | LSC |
| *L. subcostata* | p3 | **(ATT) × 4** | 12 | 59055 | 59066 |  | *acc*D-*psa*I | intergenic | LSC |
| *L. subcostata* | p3 | **(AGA) ×4** | 12 | 87890 | 87901 |  | *ycf*2 | gene | IRa |
| *L. subcostata* | p3 | **(TTA) ×5** | 15 | 111945 | 111959 |  | *ndh*F-*rpl*32 | intergenic | SSC |
| *L. subcostata* | p3 | **(ATT) ×4** | 12 | 113293 | 113304 |  | *rpl*32-*trn*L | intergenic | SSC |
| *L. subcostata* | p3 | **(TTC) ×4** | 12 | 148038 | 148049 |  | *ycf*2 | gene | IRb |
| *L. fauriei* | p4 | **(TAAG) × 3** | 12 | 42290 | 42301 |  | *ycf*3 intron 1 | intron | LSC |
| *L. fauriei* | p4 | **(AAAT) × 3** | 12 | 67867 | 67878 |  | *rpl*33-*rps*18 | intergenic | LSC |
| *L. fauriei* | p4 | **(AATA) × 3** | 12 | 74058 | 74069 |  | *psb*B-*psb*T | intergenic | LSC |
| *L. fauriei* | p4 | **(TTTC) × 3** | 12 | 74844 | 74855 |  | *psb*H-*pet*B | intergenic | LSC |
| *L. fauriei* | p4 | **(ATGT) × 3** | 12 | 77930 | 77941 |  | *rpo*A | gene | LSC |
| *L. fauriei* | p4 | **(TTTC) × 3** | 12 | 82137 | 82148 |  | *rpl*16 intron | intron | LSC |
| *L. fauriei* | p4 | **(ATAG) × 3** | 12 | 111739 | 111750 |  | *ndh*F | gene | SSC |
| *L. fauriei* | p4 | **(AATA) × 3** | 12 | 115159 | 115170 |  | *ndh*D | gene | SSC |
| *L. guilinensis* | p4 | **(TAAG) × 3** | 12 | 42385 | 42396 |  | *ycf*3 intron 1 | intron | LSC |
| *L. guilinensis* | p4 | **(AATA) × 3** | 12 | 73948 | 73959 |  | *psb*B-*psb*T | intergenic | LSC |
| *L. guilinensis* | p4 | **(TTTC) × 3** | 12 | 74734 | 74745 |  | *psb*H-*pet*B | intergenic | LSC |
| *L. guilinensis* | p4 | **(ATGT) × 3** | 12 | 77819 | 77830 |  | *rpo*A | gene | LSC |
| *L. guilinensis* | p4 | **(TTTC) × 3** | 12 | 82028 | 82039 |  | *rpl*16 intron | intron | LSC |
| *L. guilinensis* | p4 | **(ATAG) × 3** | 12 | 111515 | 111526 |  | *ndh*F | gene | SSC |
| *L. guilinensis* | p4 | **(AATA) × 3** | 12 | 114901 | 114912 |  | *ndh*D | gene | SSC |
| *L. indica* | p4 | **(TAAG) × 3** | 12 | 42382 | 42393 |  | *ycf*3 intron 1 | intron | LSC |
| *L. indica* | p4 | **(AATA) × 3** | 12 | 74186 | 74197 |  | *psb*B-*psb*T | intergenic | LSC |
| *L. indica* | p4 | **(TTTC) × 3** | 12 | 74972 | 74983 |  | *psb*H-*pet*B | intergenic | LSC |
| *L. indica* | p4 | **(ATGT) × 3** | 12 | 78055 | 78066 |  | *rpo*A | gene | LSC |
| *L. indica* | p4 | **(TTTC) × 3** | 12 | 82263 | 82274 |  | *rpl*16 intron | intron | LSC |
| *L. indica* | p4 | **(ATAG) × 3** | 12 | 111695 | 111706 |  | *ndh*F | gene | SSC |
| *L. indica* | p4 | **(AATA) × 3** | 12 | 115088 | 115099 |  | *ndh*D | gene | SSC |
| *L. indica* ‘Lüzhao Hongdie’ | p4 | **(TAAG) × 3** | 12 | 42388 | 42399 |  | *ycf*3 intron 1 | intron | LSC |
| *L. indica* ‘Lüzhao Hongdie’ | p4 | **(AATA) × 3** | 12 | 74197 | 74208 |  | *psb*B-*psb*T | intergenic | LSC |
| *L. indica* ‘Lüzhao Hongdie’ | p4 | **(TTTC) × 3** | 12 | 74983 | 74994 |  | *psb*H-*pet*B | intergenic | LSC |
| *L. indica* ‘Lüzhao Hongdie’ | p4 | **(ATGT) × 3** | 12 | 78066 | 78077 |  | *rpo*A | gene | LSC |
| *L. indica* ‘Lüzhao Hongdie’ | p4 | **(TTTC) × 3** | 12 | 82279 | 82290 |  | *rpl*16 intron | intron | LSC |
| *L. indica* ‘Lüzhao Hongdie’ | p4 | **(ATAG) × 3** | 12 | 111714 | 111725 |  | *ndh*F | gene | SSC |
| *L. indica* ‘Lüzhao Hongdie’ | p4 | **(AATA) × 3** | 12 | 115110 | 115121 |  | *ndh*D | gene | SSC |
| *L. speciosa* | p4 | **(TAAA) × 3** | 12 | 7990 | 8001 |  | *trn*S-*trn*G | intergenic | LSC |
| *L. speciosa* | p4 | **(TGTC) × 3** | 12 | 15617 | 15628 |  | *rps*2-*rpo*C2 | intergenic | LSC |
| *L. speciosa* | p4 | **(TAAG) × 3** | 12 | 42674 | 42685 |  | *ycf*3 intron 1 | intron | LSC |
| *L. speciosa* | p4 | **(AATA) × 3** | 12 | 74310 | 74321 |  | *psb*B-*psb*T | intergenic | LSC |
| *L. speciosa* | p4 | **(TTTC) × 3** | 12 | 75096 | 75107 |  | *psb*H-*pet*B | intergenic | LSC |
| *L. speciosa* | p4 | **(ATGT) × 3** | 12 | 78197 | 78208 |  | *rpo*A | gene | LSC |
| *L. speciosa* | p4 | **(TTTC) × 3** | 12 | 82411 | 82422 |  | *rpl*16 intron | intron | LSC |
| *L. speciosa* | p4 | **(ATAG) × 3** | 12 | 111970 | 111981 |  | *ndh*F | gene | SSC |
| *L. speciosa* | p4 | **(AATA) × 3** | 12 | 115318 | 115329 |  | *ndh*D | gene | SSC |
| *L. subcostata* | p4 | **(TAAG) ×3** | 12 | 42255 | 42266 |  | *ycf*3 intron 1 | intron | LSC |
| *L. subcostata* | p4 | **(AAAT) × 3** | 12 | 67836 | 67847 |  | *rpl*33-*rps*18 | intergenic | LSC |
| *L. subcostata* | p4 | **(AATA) × 3** | 12 | 74025 | 74036 |  | *psb*B-*psb*T | intergenic | LSC |
| *L. subcostata* | p4 | **(TTTC) × 3** | 12 | 74811 | 74822 |  | *psb*H-*pet*B | intergenic | LSC |
| *L. subcostata* | p4 | **(ATGT) × 3** | 12 | 77898 | 77909 |  | *rpo*A | gene | LSC |
| *L. subcostata* | p4 | **(TTTC) × 3** | 12 | 82107 | 82118 |  | *rpl*16 intron | intron | LSC |
| *L. subcostata* | p4 | **(ATAG) × 3** | 12 | 111542 | 111553 |  | *ndh*F | gene | SSC |
| *L. subcostata* | p4 | **(AATA) × 3** | 12 | 114930 | 114941 |  | *ndh*D | gene | SSC |
| *L. fauriei* | p5 | **(TCCGG) × 3** | 15 | 93570 | 93584 |  | *trn*L-*ndh*B | intergenic | IRa |
| *L. fauriei* | p5 | **(ACCGG) ×3** | 15 | 142774 | 142788 |  | *ndh*B-*trn*L | intergenic | IRb |
| *L. guilinensis* | p5 | **(TCCGG) × 3** | 15 | 93461 | 93475 |  | *trn*L-*ndh*B | intergenic | IRa |
| *L. guilinensis* | p5 | **(ACCGG) × 3** | 15 | 142529 | 142543 |  | *ndh*B-*trn*L | intergenic | IRb |
| *L. indica* | p5 | **(TCCGG) × 3** | 15 | 93696 | 93710 |  | *trn*L-*ndh*B | intergenic | IRa |
| *L. indica* | p5 | **(ACCGG) × 3** | 15 | 142541 | 142555 |  | *ndh*B-*trn*L | intergenic | IRb |
| *L. indica* ‘Lüzhao Hongdie’ | p5 | **(TCCGG) × 3** | 15 | 93712 | 93726 |  | *trn*L-*ndh*B | intergenic | IRa |
| *L. indica* ‘Lüzhao Hongdie’ | p5 | **(ACCGG) × 3** | 15 | 142567 | 142581 |  | *ndh*B-*trn*L | intergenic | IRb |
| *L. speciosa* | p5 | **(TCCGG) × 3** | 15 | 93841 | 93855 |  | *trn*L-*ndh*B | intergenic | IRa |
| *L. speciosa* | p5 | **(ACCGG) ×3** | 15 | 142864 | 142878 |  | *ndh*B-*trn*L | intergenic | IRb |
| *L. subcostata* | p5 | **(TCCGG) × 3** | 15 | 93540 | 93554 |  | *trn*L-*ndh*B | intergenic | IRa |
| *L. subcostata* | p5 | **(ACCGG) × 3** | 15 | 142385 | 142399 |  | *ndh*B intron | intron | IRb |
