# Supplementary material for: Comparative Analysis of Six Lagerstroemia Complete Chloroplast Genomes
Source: Front Plant Sci. 2017 Jan 19;8:15. doi: 10.3389/fpls.2017.00015 (PMC5243828; doi:10.3389/fpls.2017.00015)
Supplement: Supplementary file 3 [file Table3.DOCX]

**TABLE S3 | Distribution of each SSR type in each of the six *Lagerstroemia* cp genomes.**

|  | **Category** | **SSR type** | **Number** | **Intergenic** | **Gene** | **Intron** | **LSC** | **SSC** | **IRa** | **IRb** |
| --- | --- | --- | --- | --- | --- | --- | --- | --- | --- | --- |
| *L. fauriei* | Mono- nucleotide | (A)10 | 6 | 3 | 0 | 3 | 4 | 1 | 1 | 0 |
|  |  | (A)11 | 3 | 2 | 0 | 1 | 1 | 1 | 0 | 1 |
|  |  | (A)14 | 1 | 1 | 0 | 0 | 1 | 0 | 0 | 0 |
|  |  | (G)13 | 1 | 1 | 0 | 0 | 1 | 0 | 0 | 0 |
|  |  | (T)10 | 10 | 7 | 3 | 0 | 7 | 2 | 0 | 1 |
|  |  | (T)11 | 6 | 3 | 1 | 2 | 3 | 2 | 1 | 0 |
|  |  | (T)13 | 1 | 1 | 0 | 0 | 1 | 0 | 0 | 0 |
|  |  | Sub-total | 28 | 18 | 4 | 6 | 28 | 6 | 2 | 2 |
|  | Di-nucleotide | (AT)5 | 2 | 0 | 1 | 1 | 1 | 1 | 0 | 0 |
|  |  | (TA)5 | 1 | 1 | 0 | 0 | 1 | 0 | 0 | 0 |
|  |  | (TC)5 | 1 | 0 | 1 | 0 | 1 | 0 | 0 | 0 |
|  |  | Sub-total | 4 | 1 | 2 | 1 | 3 | 1 | 0 | 0 |
|  | Tri- nucleotide | (AAT)4 | 1 | 0 | 0 | 1 | 1 | 0 | 0 | 0 |
|  |  | (AGA)4 | 1 | 0 | 1 | 0 | 0 | 0 | 1 | 0 |
|  |  | (ATA)4 | 1 | 1 | 0 | 0 | 1 | 0 | 0 | 0 |
|  |  | (ATT)4 | 2 | 2 | 0 | 0 | 1 | 1 | 0 | 0 |
|  |  | (TTA)5 | 1 | 1 | 0 | 0 | 0 | 1 | 0 | 0 |
|  |  | (TTC)4 | 0 | 0 | 1 | 0 | 0 | 0 | 0 | 1 |
|  |  | Sub-total | 6 | 4 | 2 | 1 | 3 | 2 | 1 | 1 |
|  | Tetra-nucleotide | (AAAT)3 | 1 | 1 | 0 | 0 | 1 | 0 | 0 | 0 |
|  |  | (AATA)3 | 2 | 1 | 1 | 0 | 1 | 1 | 0 | 0 |
|  |  | (ATAG)3 | 1 | 0 | 1 | 0 | 0 | 1 | 0 | 0 |
|  |  | (ATGT)3 | 1 | 0 | 1 | 0 | 1 | 0 | 0 | 0 |
|  |  | (TAAG)3 | 0 | 0 | 0 | 1 | 1 | 0 | 0 | 0 |
|  |  | (TTTC)3 | 2 | 1 | 0 | 1 | 2 | 0 | 0 | 0 |
|  |  | Sub-total | 7 | 3 | 3 | 2 | 6 | 2 | 0 | 0 |
|  | Penta-nucleotide | (ACCGG)3 | 1 | 1 | 0 | 0 | 0 | 0 | 0 | 1 |
|  |  | (TCCGG)3 | 1 | 1 | 0 | 0 | 0 | 0 | 1 | 0 |
|  |  | Sub-total | 2 | 2 | 0 | 0 | 0 | 0 | 1 | 1 |
|  |  | Total | 47 | 28 | 11 | 10 | 40 | 11 | 4 | 4 |

**TABLE S3**（续）

|  | **Category** | **SSR type** | **Number** | **Intergenic** | **Gene** | **Intron** | **LSC** | **SSC** | **IRa** | **IRb** |
| --- | --- | --- | --- | --- | --- | --- | --- | --- | --- | --- |
| *L. guilinensis* | Mono- nucleotide | (A)10 | 6 | 6 | 0 | 0 | 5 | 1 | 1 | 0 |
|  |  | (A)11 | 1 | 0 | 0 | 1 | 0 | 0 | 0 | 1 |
|  |  | (G)13 | 1 | 1 | 0 | 0 | 1 | 0 | 0 | 0 |
|  |  | (T)10 | 9 | 6 | 3 | 0 | 5 | 3 | 0 | 1 |
|  |  | (T)11 | 5 | 3 | 1 | 1 | 3 | 1 | 1 | 0 |
|  |  | (T)12 | 2 | 1 | 0 | 1 | 2 | 0 | 0 | 0 |
|  |  | Sub-total | 24 | 17 | 4 | 3 | 16 | 5 | 2 | 2 |
|  | Di-nucleotide | (AT)5 | 3 | 1 | 1 | 1 | 2 | 1 | 0 | 0 |
|  |  | (TA)5 | 1 | 1 | 0 | 0 | 1 | 0 | 0 | 0 |
|  |  | (TC)5 | 0 | 0 | 1 | 0 | 1 | 0 | 0 | 0 |
|  |  | Sub-total | 4 | 2 | 2 | 1 | 4 | 1 | 0 | 0 |
|  | Tri- nucleotide | (AAT)4 | 1 | 0 | 0 | 1 | 1 | 0 | 0 | 0 |
|  |  | (AGA)4 | 1 | 0 | 1 | 0 | 0 | 0 | 1 | 0 |
|  |  | (ATA)4 | 1 | 1 | 0 | 0 | 1 | 0 | 0 | 0 |
|  |  | (ATT)4 | 2 | 2 | 0 | 0 | 1 | 1 | 0 | 0 |
|  |  | (TTA)5 | 1 | 1 | 0 | 0 | 0 | 1 | 0 | 0 |
|  |  | (TTC)4 | 1 | 0 | 1 | 0 | 0 | 0 | 0 | 1 |
|  |  | Sub-total | 7 | 4 | 2 | 1 | 3 | 2 | 1 | 1 |
|  | Tetra-nucleotide | (AATA)3 | 2 | 1 | 1 | 0 | 1 | 1 | 0 | 0 |
|  |  | (ATAG)3 | 1 | 0 | 1 | 0 | 0 | 1 | 0 | 0 |
|  |  | (ATGT)3 | 1 | 0 | 1 | 0 | 1 | 0 | 0 | 0 |
|  |  | (TAAG)3 | 1 | 0 | 0 | 1 | 1 | 0 | 0 | 0 |
|  |  | (TTTC)3 | 2 | 1 | 0 | 1 | 2 | 0 | 0 | 0 |
|  |  | Sub-total | 7 | 2 | 3 | 2 | 5 | 2 | 0 | 0 |
|  | Penta-nucleotide | (ACCGG)3 | 1 | 1 | 0 | 0 | 0 | 0 | 0 | 1 |
|  |  | (TCCGG)3 | 1 | 1 | 0 | 0 | 0 | 0 | 1 | 0 |
|  |  | Sub-total | 2 | 2 | 0 | 0 | 0 | 0 | 1 | 1 |
|  |  | Total | 44 | 27 | 11 | 7 | 28 | 10 | 4 | 4 |

**TABLE S3**（续）

|  | **Category** | **SSR type** | **Number** | **Intergenic** | **Gene** | **Intron** | **LSC** | **SSC** | **IRa** | **IRb** |
| --- | --- | --- | --- | --- | --- | --- | --- | --- | --- | --- |
| *L. indica* | Mono- nucleotide | (A)10 | 6 | 3 | 0 | 3 | 3 | 2 | 0 | 1 |
|  |  | (T)10 | 10 | 6 | 2 | 2 | 7 | 2 | 1 | 0 |
|  |  | (T)11 | 2 | 1 | 1 | 0 | 1 | 1 | 0 | 0 |
|  |  | Sub-total | 18 | 10 | 3 | 5 | 11 | 5 | 1 | 1 |
|  | Di-nucleotide | (AT)5 | 4 | 2 | 1 | 1 | 3 | 1 | 0 | 0 |
|  |  | (TA)5 | 1 | 1 | 0 | 0 | 1 | 0 | 0 | 0 |
|  |  | (TC)5 | 1 | 1 | 0 | 0 | 0 | 0 | 1 | 0 |
|  |  | Sub-total | 6 | 4 | 1 | 1 | 4 | 1 | 1 | 0 |
|  | Category | SSR type | Number | Intergenic | Gene | Intron | LSC | SSC | IRa | IRb |
|  | Tri- nucleotide | (AAT)4 | 1 | 0 | 0 | 1 | 1 | 0 | 0 | 0 |
|  |  | (AGA)4 | 1 | 0 | 1 | 0 | 0 | 0 | 1 | 0 |
|  |  | (ATA)4 | 1 | 1 | 0 | 0 | 1 | 0 | 0 | 0 |
|  |  | (ATT)4 | 2 | 2 | 0 | 0 | 1 | 1 | 0 | 0 |
|  |  | (TTA)5 | 1 | 1 | 0 | 0 | 0 | 1 | 0 | 0 |
|  |  | (TTC)4 | 1 | 0 | 1 | 0 | 0 | 0 | 0 | 1 |
|  |  | Sub-total | 7 | 4 | 2 | 1 | 3 | 2 | 1 | 1 |
|  | Category | SSR type | Number | Intergenic | Gene | Intron | LSC | SSC | IRa | IRb |
|  | Tetra-nucleotide | (AATA)3 | 2 | 1 | 1 | 0 | 1 | 1 | 0 | 0 |
|  |  | (ATAG)3 | 1 | 0 | 1 | 0 | 0 | 1 | 0 | 0 |
|  |  | (ATGT)3 | 1 | 0 | 1 | 0 | 1 | 0 | 0 | 0 |
|  |  | (TAAG)3 | 1 | 0 | 0 | 1 | 1 | 0 | 0 | 0 |
|  |  | (TTTC)3 | 2 | 1 | 0 | 1 | 2 | 0 | 0 | 0 |
|  |  | Sub-total | 7 | 2 | 3 | 2 | 5 | 2 | 0 | 0 |
|  | Category | SSR type | Number | Intergenic | Gene | Intron | LSC | SSC | IRa | IRb |
|  | Penta-nucleotide | (ACCGG)3 | 1 | 1 | 0 | 0 | 0 | 0 | 0 | 1 |
|  |  | (TCCGG)3 | 1 | 1 | 0 | 0 | 0 | 0 | 1 | 0 |
|  |  | Sub-total | 2 | 2 | 0 | 0 | 0 | 0 | 1 | 1 |
|  |  | Total | 40 | 22 | 9 | 9 | 23 | 10 | 4 | 3 |

**TABLE S3**（续）

|  | **Category** | **SSR type** | **Number** | **Intergenic** | **Gene** | **Intron** | **LSC** | **SSC** | **IRa** | **IRb** |
| --- | --- | --- | --- | --- | --- | --- | --- | --- | --- | --- |
| *L. indica* ‘Lüzhao | Mono- nucleotide | (A)10 | 8 | 5 | 0 | 3 | 6 | 1 | 1 | 0 |
| Hongdie’ |  | (A)11 | 1 | 0 | 0 | 1 | 0 | 0 | 0 | 1 |
|  |  | (C)10 | 1 | 1 | 0 | 0 | 0 | 1 | 0 | 0 |
|  |  | (G)14 | 1 | 1 | 0 | 0 | 1 | 0 | 0 | 0 |
|  |  | (T)10 | 14 | 10 | 3 | 1 | 9 | 4 | 0 | 1 |
|  |  | (T)11 | 4 | 2 | 1 | 1 | 2 | 1 | 1 | 0 |
|  |  | Sub-total | 29 | 19 | 4 | 6 | 18 | 7 | 2 | 2 |
|  | Di-nucleotide | (AT) 5 | 3 | 2 | 1 | 0 | 2 | 1 | 0 | 0 |
|  |  | (TA) 5 | 1 | 1 | 0 | 0 | 1 | 0 | 0 | 0 |
|  |  | (TC) 5 | 1 | 0 | 1 | 0 | 1 | 0 | 0 | 0 |
|  |  | Sub-total | 5 | 3 | 2 | 0 | 4 | 1 | 0 | 0 |
|  | Tri- nucleotide | (AAT) 4 | 1 | 0 | 0 | 1 | 1 | 0 | 0 | 0 |
|  |  | (AGA) 4 | 1 | 0 | 1 | 0 | 0 | 0 | 1 | 0 |
|  |  | (ATA) 4 | 1 | 1 | 0 | 0 | 1 | 0 | 0 | 0 |
|  |  | (ATT) 4 | 2 | 2 | 0 | 0 | 1 | 1 | 0 | 0 |
|  |  | (TTA) 5 | 1 | 1 | 0 | 0 | 0 | 1 | 0 | 0 |
|  |  | (TTC) 4 | 1 | 0 | 1 | 0 | 0 | 0 | 0 | 1 |
|  |  | Sub-total | 7 | 4 | 2 | 1 | 3 | 2 | 1 | 1 |
|  | Tetra-nucleotide | (AATA) 3 | 2 | 1 | 1 | 0 | 1 | 1 | 0 | 0 |
|  |  | (ATAG) 3 | 1 | 0 | 1 | 0 | 0 | 1 | 0 | 0 |
|  |  | (ATGT) 3 | 1 | 0 | 1 | 0 | 1 | 0 | 0 | 0 |
|  |  | (TAAG) 3 | 1 | 0 | 0 | 1 | 1 | 0 | 0 | 0 |
|  |  | (TTTC) 3 | 2 | 1 | 0 | 1 | 2 | 0 | 0 | 0 |
|  |  | Sub-total | 7 | 2 | 3 | 2 | 5 | 2 | 0 | 0 |
|  | Penta-nucleotide | (ACCGG) 3 | 1 | 1 | 0 | 0 | 0 | 0 | 0 | 1 |
|  |  | (TCCGG) 3 | 1 | 1 | 0 | 0 | 0 | 0 | 1 | 0 |
|  |  | Sub-total | 2 | 2 | 0 | 0 | 0 | 0 | 1 | 1 |
|  |  | Total | 50 | 30 | 11 | 9 | 30 | 12 | 4 | 4 |

**TABLE S3**（续）

|  | **Category** | **SSR type** | **Number** | **Intergenic** | **Gene** | **Intron** | **LSC** | **SSC** | **IRa** | **IRb** |
| --- | --- | --- | --- | --- | --- | --- | --- | --- | --- | --- |
| *L. speciosa* | Mono- nucleotide | (A)10 | 5 | 5 | 0 | 0 | 5 | 0 | 0 | 0 |
|  |  | (A)11 | 1 | 0 | 0 | 1 | 0 | 0 | 0 | 1 |
|  |  | (A)14 | 1 | 1 | 0 | 0 | 1 | 0 | 0 | 0 |
|  |  | (C)12 | 1 | 0 | 0 | 1 | 1 | 0 | 0 | 0 |
|  |  | (G)10 | 1 | 1 | 0 | 0 | 1 | 0 | 0 | 0 |
|  |  | (T)10 | 11 | 7 | 2 | 2 | 9 | 2 | 0 | 0 |
|  |  | (T)11 | 3 | 2 | 0 | 1 | 2 | 0 | 1 | 0 |
|  |  | (T)13 | 1 | 1 | 0 | 0 | 1 | 0 | 0 | 0 |
|  |  | Sub-total | 24 | 17 | 2 | 5 | 20 | 2 | 1 | 1 |
|  | Di-nucleotide | (AT)5 | 2 | 1 | 1 | 0 | 2 | 0 | 0 | 0 |
|  |  | (AT)6 | 1 | 0 | 0 | 1 | 0 | 1 | 0 | 0 |
|  |  | (TA)5 | 2 | 2 | 0 | 0 | 1 | 1 | 0 | 0 |
|  |  | (TC)5 | 1 | 0 | 1 | 0 | 1 | 0 | 0 | 0 |
|  |  | Sub-total | 6 | 3 | 2 | 1 | 4 | 2 | 0 | 0 |
|  | Tri- nucleotide | (AAT)4 | 1 | 0 | 0 | 1 | 1 | 0 | 0 | 0 |
|  |  | (AGA)4 | 1 | 0 | 1 | 0 | 0 | 0 | 1 | 0 |
|  |  | (ATA)4 | 1 | 1 | 0 | 0 | 1 | 0 | 0 | 0 |
|  |  | (ATT)4 | 2 | 2 | 0 | 0 | 1 | 1 | 0 | 0 |
|  |  | (TTA)4 | 1 | 1 | 0 | 0 | 1 | 0 | 0 | 0 |
|  |  | (TTC)4 | 1 | 0 | 1 | 0 | 0 | 0 | 0 | 1 |
|  |  | Sub-total | 7 | 4 | 2 | 1 | 4 | 1 | 1 | 1 |
|  | Tetra-nucleotide | (AATA)3 | 2 | 1 | 1 | 0 | 1 | 1 | 0 | 0 |
|  |  | (ATAG)3 | 1 | 0 | 1 | 0 | 0 | 1 | 0 | 0 |
|  |  | (ATGT)3 | 1 | 0 | 1 | 0 | 1 | 0 | 0 | 0 |
|  |  | (TAAA)3 | 1 | 1 | 0 | 0 | 1 | 0 | 0 | 0 |
|  |  | (TAAG)3 | 1 | 0 | 0 | 1 | 1 | 0 | 0 | 0 |
|  |  | (TGTC)3 | 1 | 1 | 0 | 0 | 1 | 0 | 0 | 0 |
|  |  | (TTTC)3 | 2 | 1 | 0 | 1 | 2 | 0 | 0 | 0 |
|  |  | Sub-total | 9 | 4 | 3 | 2 | 7 | 2 | 0 | 0 |
|  | Penta-nucleotide | (ACCGG)3 | 1 | 1 | 0 | 0 | 0 | 0 | 0 | 1 |
|  |  | (TCCGG)3 | 1 | 1 | 0 | 0 | 0 | 0 | 1 | 0 |
|  |  | Sub-total | 2 | 2 | 0 | 0 | 0 | 0 | 1 | 1 |
|  |  | Total | 48 | 30 | 9 | 9 | 35 | 7 | 3 | 3 |

**TABLE S3**（续）

|  | **Category** | **SSR type** | **Number** | **Intergenic** | **Gene** | **Intron** | **LSC** | **SSC** | **IRa** | **IRb** |
| --- | --- | --- | --- | --- | --- | --- | --- | --- | --- | --- |
| *L. subcostata* | Mono- nucleotide | (A)10 | 4 | 2 | 0 | 2 | 2 | 1 | 1 | 0 |
|  |  | (A)11 | 2 | 1 | 0 | 1 | 1 | 0 | 0 | 1 |
|  |  | (A)15 | 1 | 1 | 0 | 0 | 1 | 0 | 0 | 0 |
|  |  | (G)10 | 1 | 1 | 0 | 0 | 1 | 0 | 0 | 0 |
|  |  | (T)10 | 12 | 8 | 3 | 1 | 7 | 4 | 0 | 1 |
|  |  | (T)11 | 3 | 1 | 1 | 1 | 1 | 1 | 1 | 0 |
|  |  | (T)12 | 1 | 0 | 0 | 1 | 1 | 0 | 0 | 0 |
|  |  | (T)14 | 1 | 1 | 0 | 0 | 1 | 0 | 0 | 0 |
|  |  | Sub-total | 25 | 15 | 4 | 6 | 15 | 6 | 2 | 2 |
|  | Di-nucleotide | (AT) 5 | 2 | 0 | 1 | 1 | 1 | 1 | 0 | 0 |
|  |  | (TA) 5 | 1 | 1 | 0 | 0 | 1 | 0 | 0 | 0 |
|  |  | (TC) 5 | 1 | 0 | 1 | 0 | 1 | 0 | 0 | 0 |
|  |  | Sub-total | 4 | 1 | 2 | 1 | 3 | 1 | 0 | 0 |
|  | Tri- nucleotide | (AAT) 4 | 1 | 0 | 0 | 1 | 1 | 0 | 0 | 0 |
|  |  | (AGA) 4 | 1 | 0 | 1 | 0 | 0 | 0 | 1 | 0 |
|  |  | (ATA) 4 | 1 | 1 | 0 | 0 | 1 | 0 | 0 | 0 |
|  |  | (ATT) 4 | 2 | 2 | 0 | 0 | 1 | 1 | 0 | 0 |
|  |  | (TTA) 5 | 1 | 1 | 0 | 0 | 0 | 1 | 0 | 0 |
|  |  | (TTC)4 | 1 | 0 | 1 | 0 | 0 | 0 | 0 | 1 |
|  |  | Sub-total | 7 | 4 | 2 | 1 | 3 | 2 | 1 | 1 |
|  | Tetra-nucleotide | (AAAT) 3 | 1 | 1 | 0 | 0 | 1 | 0 | 0 | 0 |
|  |  | (AATA) 3 | 2 | 1 | 1 | 0 | 1 | 1 | 0 | 0 |
|  |  | (ATAG) 3 | 1 | 0 | 1 | 0 | 0 | 1 | 0 | 0 |
|  |  | (ATGT) 3 | 1 | 0 | 1 | 0 | 1 | 0 | 0 | 0 |
|  |  | (TAAG) 3 | 1 | 0 | 1 | 0 | 1 | 0 | 0 | 0 |
|  |  | (TTTC) 3 | 2 | 1 | 0 | 1 | 2 | 0 | 0 | 0 |
|  |  | Sub-total | 8 | 3 | 4 | 1 | 6 | 2 | 0 | 0 |
|  | Penta-nucleotide | (ACCGG) 3 | 1 | 0 | 0 | 1 | 0 | 0 | 0 | 1 |
|  |  | (TCCGG) 3 | 1 | 1 | 0 | 0 | 0 | 0 | 1 | 0 |
|  |  | Sub-total | 2 | 1 | 0 | 1 | 0 | 0 | 1 | 1 |
|  |  | Total | 46 | 24 | 12 | 10 | 27 | 11 | 4 | 4 |
